# Identification of spoilage microorganisms in blueberry juice and their inactivation by a microchip pulsed electric field system

**DOI:** 10.1038/s41598-018-26513-2

**Published:** 2018-05-25

**Authors:** Ning Zhu, Ning Yu, Yue Zhu, Yulong Wei, Yanan Hou, Haiping Zhang, Ai-dong Sun

**Affiliations:** Department of Food Science and Engineering, College of Biological Sciences and Technology, Beijing Forestry University, Beijing, 100083 China

## Abstract

Blueberry juice is a healthy and nutritious food that has become increasingly popular worldwide. However, little is known about the microbial groups of this juice that can cause its spoilage. This study aimed to identify the main spoilage microorganisms in blueberry juice and explore whether a microchip pulsed electric field (MPEF) can effectively inactivate them. We performed polymerase chain reaction (PCR) amplification, as well as 16S rDNA, 18S rDNA, internal transcribed spacer (ITS), and 26S rDNA gene sequence analyses. Nine species belonging to eight genera, including *Pantoea*, *Burkholderia*, *Pichia*, *Meyerozyma*, *Cryptococcus*, *Aureobasidium*, *Cladosporium*, and *Penicillium* were identified as spoilage microorganisms. *Cryptococcus* sp., *Meyerozyma* sp., and *Pichia* sp. were specific spoilage organisms (SSO) owing to their rising numbers throughout spoilage progression. The effect of MPEF on the potential inactivation of these microorganisms was to induce significant inactivation of viable *Cryptococcus* sp., *Meyerozyma* sp., and *Pichia* sp. This research provides a theoretical basis for the application of MPEF in improving the quality of blueberry juice.

## Introduction

Fruits juices are highly popular because of their rich nutrition and high moisture. These features simultaneously provide a good environment for microbial growth, leading to the spoilage of fruit juice during storage and circulation^1^. However, not all microorganisms have an effect on spoilage, as Koch verified regarding the relationship between plant disease and viruses, and this principle of course also applies to other fields^2^, including spoilage and the identification of spoilage microorganisms. Dalgaard^3^ put forward the concept of specific spoilage organisms (SSO) in 1995, referring to those microbes accounting for relatively small percentage in fresh products, but growing faster than other microorganisms during storage and producing putrid odour. Özogul^4^ pointed out that the growth and reproduction of SSO dominate food spoilage to a large extent. Thus, thoroughly understanding the SSO and new sterilization methods are important to effectively reduce the health risks caused by spoilage microorganisms^5^.

Blueberry contains a variety of trace and mineral^6^ elements that have various physiological functions^7–9^ in human physiology owing to the fruit’s high polyphenol content^10^. A major challenge for the blueberry industry is the short shelf life of blueberries due to the seasonal susceptibility of blueberries to microbial spoilage. Edible coatings have been used to extend shelf life of blueberries, such as quinoa protein, chitosan, sunflower oil^11^, liposomes containing d-limonene^12^ and chitosan–*Aloevera*^13^. Cold plasma can inactivate spoilage microorganisms of blueberries without a need for temperature changes^14^. Water-assisted pulsed light treatment^15^ is also an effective way to inactivate microorganisms on blueberries, which have minimal impact on quality attributes. Furthermore, some studies have validated the bactericidal effect of chlorine dioxide gas^16^, and the combination of ultraviolet light and ozone^17^ for reducing the spoilage of blueberries. These methods are mainly applied to the preservation of blueberry fruit. Meanwhile, blueberry juice is a popular beverage and accounts for a large part of the juice market. However, research on blueberry juice is mainly focused on its properties and function^18,19^. Thus far, there have been no studies about the identification of spoilage microorganisms in blueberry juice.

Research efforts have recently focused on the sterilization of pulsed electric fields for liquid foods^20–23^. However, microbial inactivation generally requires a strong electric field generated by a very high voltage ranging from several kilovolts to tens of kilovolts^24^. This process leads to high costs and electrolysis-prone areas around electrodes. With the development of microfabrication, in which the space between two electrodes is short, low voltage can produce high electric field strength. Functional materials with microstructures have attract increasing interesting^25,26^, to date, several laboratories have developed microchips with germicidal function^27^. Whether this new technology can be used to inactivate spoilage microorganisms in blueberry juice remains to be verified.

In the present study, we used morphology^28^, 16S rDNA, 18S rDNA, 26S rDNA, and ITS sequence analyses to identify the microorganisms presenting blueberry juice and those causing blueberry juice spoilage, and then constructed phylogenetic trees using MEGA 5.0 Software. SSO in blueberry juice were confirmed by investigating any correlations between changing numbers of microorganisms and spoilage with time. Finally, the effects of inactivation on spoilage microorganisms under MPEF were investigated. The aim of this research is to determine the native of SSO in blueberry juice and verify whether MPEF can effectively inactivate them. Our results provide a reference for the further development of the blueberry juice processing industry.

## Results

### Isolation and verification of spoilage microorganisms in blueberry juice

The degree of spoilage of blueberry juice in different storage days was evaluated through comprehensive comparison of muddy appearance, flocculent precipitate, acid taste and total microbial numbers. As shown in Table 1, the juice gradually became turbid with time, the total number of dominant microorganisms and acidity both increased faster confirmed by decreased pH values, and there was an increasing trend in conductivity with time, as spoilage progressed. Isolations were carried out by picking colonies from each culture medium on the basis of their different morphologies. Twenty-four different candidate spoilage microbial species were isolated from blueberry juice (0–3 d), isolates 00, 01 were isolated on day 0; 10–19 were isolated on day 1; 20–24 were isolated on day 2; 30–36 were separated on day 3. Among them, 00, 10, 11, 20, 30 were isolated and purified from LB medium; 13, 14, 15, 16, 17, 18, 19, 23, 24, 33, 34, 35, 36 were isolated and purified from Bengal red agar medium; 01, 22, 32 were isolated and purified from MSA medium; 12, 21, 31 were isolated and purified from MRS medium.

**Table 1:** Comparison of microbial load and spoilage of blueberry juice in storage days at 25 °C.

Days (d)	Number of different genera	Muddy	Flocculent precipitate	Acidity by smell	Total number of colonies (lg cfu/mL)	pH	Conductivity	Degrees of spoilage
0	2	—	—	—	2.20 ± 0.07	3.35	212	—
1	10	+	+	++	5.91 ± 0.12	3.17	229	+
2	5	++	++	++	7.15 ± 0.09	3.08	254	++
3	7	++	++	++	7.01 ± 0.15	3.07	258	++

Note: − means no muddy, precipitate, or acid; + means muddy, precipitate, or acid; a more number of + means higher intensity of muddy, precipitate, or acid, which means more serious degree of spoilage.

Purified microorganisms were inoculated into test tubes of sterilized blueberry juice; most of them had no obvious change after 12 h, except for the reduction in transparency by strains 13, 18, 23, and 34 tubes compared with the control group. The transparency using strains 12, 14, 16, 19, 21, 24, 31, 35, and 36 tubes decreased and became acidic by smell at the same time. Juice with strains 11 and 15 tubes started to smell peculiarly. Strains 13, 18, 23, and 34 produced juice that was muddier and had a rancid odor after 24 h. Strains 32 tubes became muddy and acidic after 36 h. The levels of spoilage of the tubes became more serious with prolonged culture time. However, less obvious differences were observed between juices with strains 00, 01, 10, 17, 20, 22, 30, 33, and the control juice, meaning that these strains are not causing blueberry juice spoilage. These spoilage isolates 11, 12, 13, 14, 15, 16, 18, 19, 21, 23, 24, 31, 32, 34, 35, and 36 were inoculated back into corresponding media, and the original microorganisms were recovered once again. Thus, these microorganisms are the causative agents of blueberry juice spoilage according to Koch’s postulates. In accordance with the juice safety standards in China, the edible juices have no peculiar smell, and the total number of colonies should be less than 10^2^ CFU/mL. After 72 hours culturing, the control group kept its original color, smell, and transparency, whereas the others showed different degrees of spoilage, and the total number of colonies in these groups exceeded permitted levels^29^.

The colony characteristics and cell morphology of the spoilage microorganisms are shown in Table 2.

**Table 2:** Morphological characteristics of 16 different isolates.

Strain	Colony characteristics	Cell morphology	Gram stain
11	The edge of colony is irregular, the yellow wetted surround, the white drying inside, and white hyphae seemed to grow like the radial epitaxial growth.	rod-like, small	−
12	The edge of colony is regular, yellow, long, bumpy, and viscous around.	rod-like, large	/
13	The edge of colony is regular, pink, white edge, smooth surface, and has a wet overall state.	spherical, larger, capsule around colony	+
14	The edge of colony is regular, round, deep red, smooth surfaced, bumpy, cheese-like, and opaque	oval or elliptical, large	/
15	The edge of colony is regular, round, bumpy, mildly myxoid, and opaque.	rod-like, amastigotes	−
16	The edge of colony is irregular, pink around, deep red in the middle, and mildly myxoid.	elliptical, darkened color at later stage	/
18	The edge of colony is irregular, roundish, light green around, white in the middle, filamentous, dry and opaque.	long rod-like, large	/
19	The edge of colony is irregular, deep colored, dry and opaque.	long oval, large	/
21	The edge of colony is regular, round, beige, long, bumpy, and the surroundings are mildly myxoid.	long rod-like, large	/
23	The edge of colony is regular, round, pink, white edged, smooth surfaced, and has a wet overall state.	spherical, large, capsule around colony	+
24	The edge of colony is regular, round, deep red, smooth surfaced, bumpy, cheese-like, and opaque.	oval or elliptical, large	/
31	The edge of colony is regular, round, beige, long, bumpy, and the surroundings are mildly myxoid.	rod-like, large	/
32	The edge of colony is regular, round, deeply colored around, lightly colored in the middle, bumpy, mildly myxoid in the edge.	long rod-like, large	/
34	The edge of colony is regular, round, pink, white edged, smooth surfaced, and has a wet overall state	spherical, large, capsule around colony	+
35	The edge of colony is regular, round, long, bumpy, and the surroundings are mildly myxoid.	rod-like, large	/
36	The edge of colony is regular, round, deep red, smooth surfaced, bumpy, cheese-like, and opaque.	oval or elliptical, large	/

Notes: + Gram stain positive; − Gram stain negative; / Not determined.

As shown in Table 2, strains 12, 21, 31 and 35 had similarities regarding rod-like shape, regular edge colonies, and so on. Strains 13, 23, 34 shared similar pink colored colonies, spherical shape cells, regular edge, smooth surface colonies. Strains 14, 24, 36 had similar red colored colonies, oval shape cells, regular edge, smooth surface, cheese-like colonies. Therefore, microscopic and macroscopic observation suggested the following: strains 12, 21, 31 and 35; strains 13, 23 and 34; strains 14, 24 and 36 were similar and could be related species, which as also verified by the PCR. Following the growing of related microorganisms, nine representations of the spoilage microorganisms were identified for further study. 11, 12, 13, 14, 15, 16, 18, 19, and 32 were identified as the microorganisms that can cause spoilage of blueberry juice.

### Molecular identification of SSO in blueberry juice

DNA was extracted with SK 8255 kit for strain 11, SK8259 kit for strain 18, and SK8257 kit for strains 16 and 19. In addition, DNA was extracted with D3350–01 kit for strain 15, D3390-01 kit for strains 12, 13, and 32, and D3370-01 kit for strain 14. All the electrophoresis results showed clear, single, stable strips. The description and similarities of the nine microorganisms are shown in Table 3 according to the results of sequence alignment and phylogenetic trees.

**Table 3:** Identification of spoilage microorganisms using sequence analysis.

strain	Description	Similarities	E-value
11	*Pantoea eucalypti* (NR116112)	99%	0
15	*Burkholderia fungorum* (NR114118)	99%	0
18	*Penicillium purpurogenum* (AF245255)	99%	0
12	*Meyerozyma caribbica* (AB032175)	100%	0
13	*Cryptococcus laurenti* (JN627008)	100%	0
32	*Meyerozyma guilliermondii* (HM535382)	100%	0
14	*Pichia guilliermondii* (EF063127)	99%	0
16	*Aureobasidium pullulans* (KT922591)	99%	0
19	*Cladosporium ramotenellum* (KM521834)	100%	0

By analyzing the 16S rDNA sequence comparison results, strain 11 corresponded to *Pantotea* sp., and strain 15 corresponded to *Burkholderia* sp. At the same time, the phylogenetic tree showed that strain 11 was most closely related to the *Pantoea eucalypti* (99% 16S rDNA similarity), so it was identified as *Pantoeai* sp., and strain 15 was most closely related to the *Burkholderia fungorum* (99% 16S rDNA similarity), so it was identified as *Burkholderia* sp. The comparison results were consistent with the analysis result of the phylogenetic tree. The closest match for fungus 18 in the 18S rDNA analysis was *Penicillium purpurogenum*. E values were zero, which meant it was full match, and the similarity level is up to 99%. This result was consistent with the analysis result of phylogenetic tree, so strain 18 was classified as genus *Penicillum*, and the closest matching microorganism was *Penicillium purpurogenum* (Table 3).

The highest homology of the ITS sequence of fungus 12 was with *Meyerozyma caribbica* (AB032175), fungus 13 was with *Cryptococcus laurenti* (JN627008) and fungus 32 was with *Meyerozyma guilliermondii* (HM535382). There were all 100% matches. Sequence similarities that exceed 97% are considered to be the same species^30^, those <96–97% are considered to be different species, and those <93–95% are considered to be different genera^31^. Thus, according to this scheme, strains 12 and 32 belonging to the genus *Meyerozyma* and strain 13 to the genus *Cryptococcus*.

Strain 14 had the highest homology (up to 99%) with *Pichia guilliermondii*, strain 16 exhibited 99% homology with *Aureobasidium pullulans*, and strain 19 had 100% homology with *Cladosporium ramotenellum*. The results of phylogenetic trees were consistent with the comparison results. Therefore, isolates 14, 16 and 19 were identified as *Pichia* sp., *Aureobasidium* sp. and *Cladosporium* sp., respectively.

**Figure 1:**
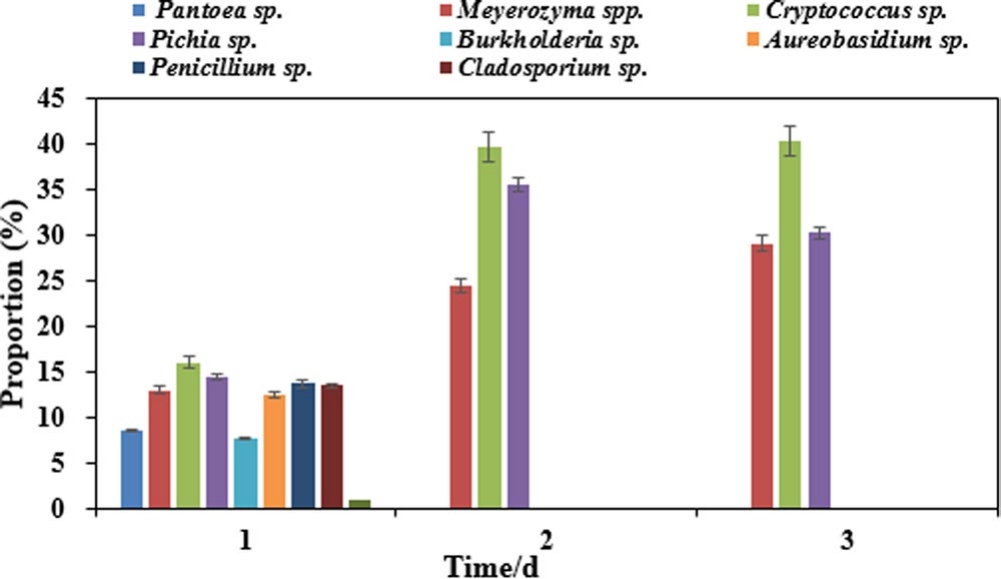
Change trend of spoilage isolates at room temperature. Different colors of the columns represent the percentages of remaining microorganisms in each days.

Figure 1 shows the changing trend of the major spoilage microorganisms in fresh blueberry juice at room temperature with time. Initially (day 1), the microbial flora was complex, and all 8 species were present in detectable proportion (8–16%). The proportion of *Pantoea* sp., *Meyerozyma* spp., *Cryptococcus* sp., *Pichia* sp., *Burkholderia* sp., *Aureobasidium* sp., *Penicillium* sp., and *Cladosporium* sp. were 8.7%, 13.1%, 16.1%, 14.5%, 7.7%, 12.5%, 13.8%, and 13.6%, respectively. Thus, *Cryptococcus* sp. accounted for the largest proportion, followed by *Pichia* sp. and *Penicillium* sp. The relative proportion changed by day 2, the proportion of *Meyerozyma* sp., *Cryptococcus* sp., *Pichia* sp. were significantly increased, and few other genera were detected. Thereafter, the proportions of spoilage microorganisms exhibited little further change, *Cryptococcus* sp. accounted for the largest proportion at 40.50%, and the proportion of *Meyerozyma* sp., and *Pichia* sp. tended to be appearently equally balance (about 30%). Therefore, *Cryptococcus* sp., *Meyerozyma* sp., and *Pichia* sp. were the predominant SSO in the process of blueberry juice spoilage.

### Effect of MPEF processing on spoilage microorganisms

**Figure 2:**
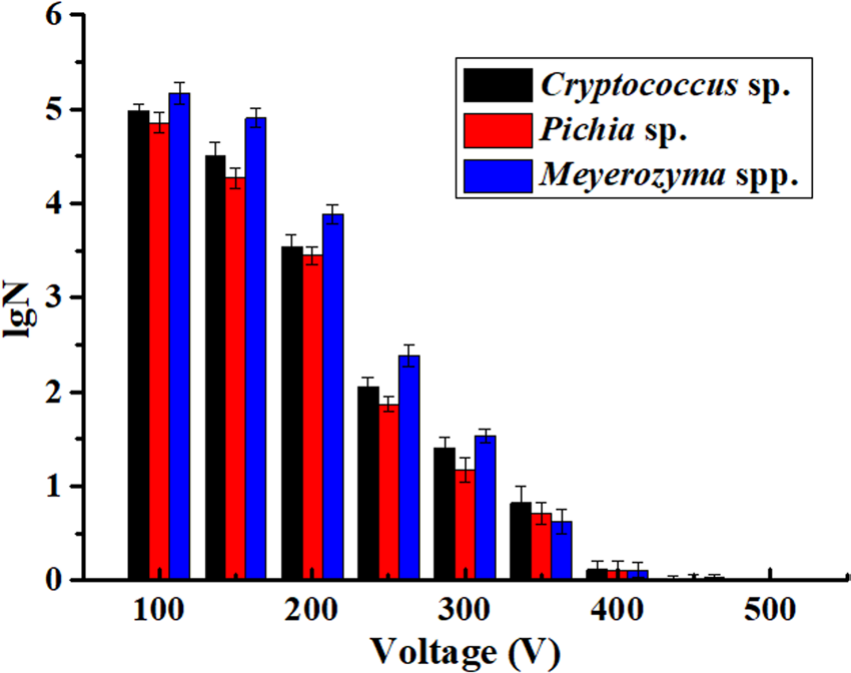
Inactivation effect of SSO in different pulse voltage. Different colors of the columns represent the logarithm of viable cell after MPEF treatment.

Original concentrations of *Cryptococcus* sp., *Pichia* sp. and *Meyerozyma* sp. in blueberry juice were 5.93 ± 0.11, 5.86 ± 0.09 and 6.08 ± 0.10 lg CFU/mL. Figure 2 shows the influence of voltage on subsequent viability of each SSO after MPEF treatment. Black, red, and violet histograms represent the variation in logarithmic survival of *Cryptococcus* sp., *Pichia* sp., and *Meyerozyma* spp., respectively.

A significant (*P* < 0.05) effect of voltage was observed (4.98 ± 0.08 to 0 log_10_ cycles for *Cryptococcus* sp.) as the operating voltage was changed from 100 V to 500 V, thereby indicating the efficacy of voltage in reducing microbial counts (Fig. 2). Simultaneously, the viable number of *Pichia* sp. and *Meyerozyma* spp. decreased 4.85 ± 0.11 and 5.16 ± 0.12 log_10_ cycles, respectively. This result showed that MPEF significantly affected SSO viability; however, the decline in microbial counts was much less pronounced as voltage increased from 400 V to 500 V. Thus, 400 V was selected to study the inactivation effect of other spoilage microorganisms in blueberry juice (Table 4), and pulse duration is set to 0.2 ms. *lgN*_0_ was the logarithm of microorganisms before treatment, and *lgS* was the logarithmic decrease after treatment. Results indicated that this treatment can significantly reduce and effectively inactivate spoilage microorganisms in blueberry juice.

**Table 4:** Inactivation of spoilage microorganisms at a pulse voltage of 400 V.

Strain	*Pantoea* sp.	*Burkholderia* sp.	*Aureobasidium* sp.	*Cladosporium* sp.	*Penicillium* sp.
*lgN*_0_	5.82 ± 0.04	5.24 ± 0.06	5.77 ± 0.05	5.63 ± 0.06	5.52 ± 0.05
*lgS*	5.15 ± 0.13	5.09 ± 0.11	4.68 ± 0.15	4.42 ± 0.15	4.31 ± 0.16

## Discussion

Most previous studies carried out on blueberry juice have considered only functional and sensory quality of blueberry juice^32^. The identification of SSO and their inactivation by MPEF is novel, and to our knowledge, not previously been investigated yet, identifying spoilage microorganisms is necessary to effectively improve the quality of juice.

Firstly, 24 candidate spoilage microorganisms were isolated and purified from blueberry juice. Among them, eight strains (00, 01, 10, 17, 20, 22, 30, 33) were validated that cannot cause detectable spoilage, and they may occur in the juice as a result of coincidental contamination^33^ via cleaning, squeezing or other steps in blueberry handling processing. Secondly, strains 12, 21, 31, and 35; strains 13, 23, and 34; strains 14, 24 and 36 were the same genera through microscopic observation and PCR. Finally, nine strains that led to blueberry juice spoilage were identified.

PCR amplification^34^ of the nine spoilage strains was further carried out. Results showed that the spoilage of blueberry juice was caused by two bacterial genera: *Pantoea* (11) and *Burkholderia* (15), four species of yeast belonging to three genera: *Pichia* (14), *Cryptococcus* (13), and *Meyerozyma* (12, 32), and three fungal genera: *Aureobasidium* (16), *Penicillium* (18), and *Cladosporium* (19). During storage, the number of *Cryptococcus* sp., *Meyerozyma* spp., and *Pichia* sp. increased, while others reduced to undetectable level, and these events were accompanied by increased degree of spoilage of blueberry juice. Therefore, *Cryptococcus* sp., *Meyerozyma* spp., and *Pichia* sp. were identified as SSO.

Meiling *et al*.^35^ isolated *Pantoea* sp. from canned fruit and confirmed that it is the main microorganism in the process of spoilage. Nayeri *et al*.^36^ indicated that one of the main microorganisms that leads to juice spoilage is *Penicillium* sp. Sancho *et al*.^37^ reported that *Cryptococcus* sp. can lead to the spoilage of pineapple, orange, peach, and pear juices. *Meyerozyma guilliermondii* has been used in a dough-fermentation process by the report of Coda *et al*.^38^. Many studies have focused on spoilage caused by *Pichia* sp., which is the usual spoilage microorganism in juice, and our results are consistent with those^39^. Stratford^40^ also indicated that *Pichia* sp. is the common putrefying microorganism in food. Eight strains of *Pichia* sp. have been separated from spoilage orange juice by Qing^41^. *Pichia* sp. has been isolated from spoiled wine by Saez *et al*.^42^, and it was confirmed to be the main microorganism which caused spoilage. *Cladosporium* sp. is also one of the usual microorganisms that cause juice spoilage; Yue^43^ isolated this microorganism from spoiled blueberries and determined its spoilage behavior. All these studies showed that the microorganisms we isolated with present study are commonly found in fruits and juice, and they can cause different forms of food spoilage.

In summary, we have shown that blueberry juice is prone to spoilage due to microbial activity (Table 1). We have also shown that high pulsed electric fields can inactivate microbial growth^44^, however, to achieve the same field strength, a voltage that is 25 times higher than MPEF is required in the PEF processing system^26^. In our experiments, the inactivation effect of SSO by MPEF was enhanced with increasing voltage, which is consistent with previous findings^45,46^. Different microorganisms exhibited varied degree of sensitivity to the electric field based on a comparison of logarithmic reduction at 400 V. The most sensitive strain was yeast (*Cryptococcus* sp., *Pichia* sp., and *Meyerozyma* spp.), followed by bacteria (*Pantoea*and sp., *Burkholderia* sp.). Fungal species (*Aureobasidium* sp., *Cladosporium* sp., and *Penicillium* sp.) demonstrated the strongest resistance to the electric field. Thus, a higher voltage was required for a better inactivation effect for fungus^47^. Moreover, MPEF was more beneficial to the inactivation of SSO than other spoilage microorganisms in blueberry juice and can be used for blueberry juice sterilization.

## Conclusions

This study contributed to knowledge of SSO in blueberry juice and provided first insight into the inactivation of spoilage microorganisms by MPEF. Results indicated that only 9 out of 24 isolated and purified species can lead to blueberry juice spoilage based on microbiology and molecular identification methods. *Cryptococcus* sp., *Meyerozyma* spp., and *Pichia* sp. were the SSO in blueberry juice. Spoilage microorganisms from blueberry juice were inactivated by MPEF, and a lower voltage was needed to inactivate SSO compared with other spoilage microorganisms.

This study can provide theoretical support for preservation technology and better guarantee the quality of blueberry juice. However, further experiments are needed to identify all microorganisms that cause blueberry juice spoilage, because some microorganisms may not be effectively separated through the plate method. Moreover, the effects of different sterilization methods on the inactivation of spoilage microorganisms and the quality of juice can also be compared to determine the optimum blueberry juice sterilization method and improve its quality.

## Materials and Methods

### Isolation and purification of spoilage microorganisms

Blueberries provided by Organic Food Co., Ltd (Dandong City, Liaoning Province, China) were squeezed (1:6 with water), centrifuged, filtered, and then divided into four groups. They were stored at room temperature for 0, 1, 2 and 3 days, respectively. The total number of isolates of different groups were calculated using data obtained on plate count agar (24 h at 37 °C).

Blueberry juice samples (1 mL) stored for different days were collected and added to 15–20 mL sterilized Luria–Bertani (LB), Bengal red agar, manitol salt agar (MSA), De Man, Rogosa, Sharpe (MRS) medium^48^ and then those media were used for the isolation to select for total bacteria, total fungi, Staphylococcus and Micrococcaceae species, and lactic acid bacteria, respectively. Inoculated LB and MRS were cultivated at 37 °C for 24 h, MSA was cultivated at 30 °C for 48 h, Bengal red agar was cultivated at 28 °C for 5 days. In order to obtain pure isolates, single colonies were picked and further grown on the corresponding media at least once. These candidate isolates were preserved on an inclined plane at 4 °C.

### Verification of spoilage microorganisms in blueberry juice

Blueberry juice was sterilized at 95 °C for 15S (High temperature short time, HTST), and the content of vitamin C was decreased from 41.95 ± 0.61 μg/mL in fresh juice to 34.20 ± 0.75 μg/mL in sterilized juice. At the same time, total phenols, titratable acid and soluble reducing sugar exhibited no significant changes. The conductivity of blueberry juice was measured by Conductivity Meter (WTW, Cond 330i). Candidate isolates were inoculated in a tube of sterilized juice, and cultured at 28 °C. Changes in sensory information of the juice were recorded every 12 h, and the degree of spoilage in different tubes was recorded during 72 h by appearance (muddy, transparency), acidity and smell.

### Morphological observation

Slides were prepared for the spoilage microorganisms, isolates were initially differentiated by the differences in degree of colony smoothness, color, size, shape, edge, transparency of colonies. Growth from purified cultures were observed by microscopy (Olympus IX71) at a magnification.

### Molecular identification of microorganisms

#### DNA extraction

DNA was extracted directly from strains using a DNA Isolation Kit according to the manufacturer’s instructions. PCR was performed with the universal bacterial primers 27 F(5′-AGTTTGATCMTGGCTCAG-3′) and 1492 R (5′-GGTTACCTTGTTACGACTT-3′). NS1 (5′-GTAGTCATATGCTTGTCTC-3′) and NS6 (5′-GCATCACAGACCTGTTATTGCCTC-3′) primers were used to amplify the 18S gene. ITS1 (5′-TCCGTAGGTGAACCTGCGG-3′) and ITS2 (5′-TCCTCCGCTT A TTGA TA TGC-3′) primers were used to amplify the ITS gene. 26S rRNA gene was amplified with primers NL1(5′-GCATATCAATAAGCGGAGGAAAAG-3′) and NL4(5′-GGTCCGTGTTTCAAGACGG-3′).

#### PCR conditions

PCR amplification was carried out in a final volume of 50 μl comprising 0.5 μl of template (genome DNA 20–50 ng/μl), 2.5 μl of 10 × buffer (with M^2+^), 1 μl of dNTP (2.5 mM), 0.2 μl of enzyme, 0.5 μl of primer F (10 µM), 0.5 μl of primer R (10 µM), and 25 μl of dd H_2_O. The reactions were carried out in a Gene Amp PCR System 2720 (Applied Biosystems). Samples were incubated for 4 min at 94 °C and then cycled 30 times at 94 °C for 45S, 55 °C for 45S and 72 °C for 1 min. The samples were incubated for 10 min at 72 °C for repair and extension and stored at 4 °C until termination of reaction. Electrophoresis was performed with 1% agarose gels at 150 V and 100 mA for 20 min. One band from each sample was excised and purified with a DNA kit (EG01 Kit) according to the manufacturer’s instructions.

#### Similarity analysis

The sequencing results of the above PCR products were analyzed using Bio Edit and DNAMAN software, and then 16S rDNA sequences were compared in the Ribosomal Data Base (http://rdp.cme.msu.edu/index.jsp). ITS sequences were subjected to homology analysis on www.ezbiocloud.net; the rest were identified by aligning the obtained gene sequences with GenBank using BLAST. Finally, the reference sequences were downloaded and the phylogenetic trees were constructed with MEGA 5.0 software. The MEGA 5.0 online database provides statistical methods for molecular evolution, including building sequence alignments and phylogenetic trees^49^.

### Analysis of changes in spoilage microorganisms in blueberry juice

The numbers of each genus of spoilage microorganism in different storage days were determined, their percentage were calculated, and the changes in their presence with time during spoilage were determined^50^.

### Inactivation effect of MPEF on spoilage microorganisms

**Figure 3:**
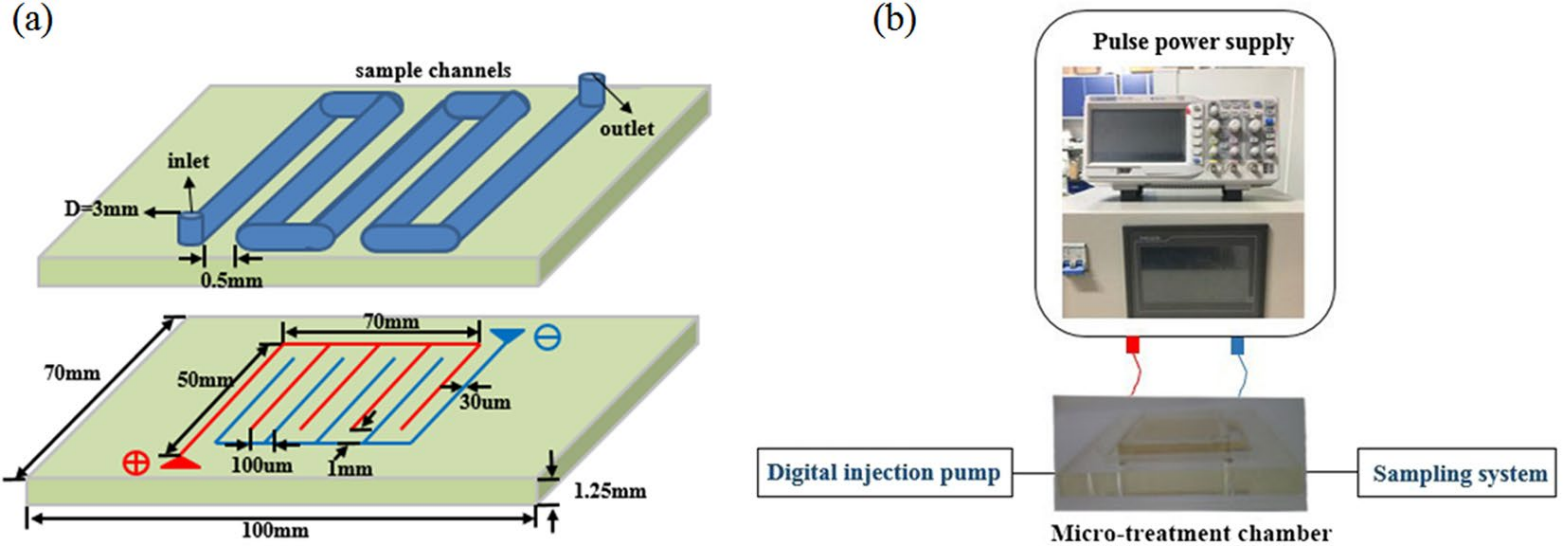
Schematic of the microchip (**a**) and experimental setup (**b**). In Fig. 3a, the upper plane shows the channel of the microchip and the lower plane represents the electric shock. The real microchip with the experiment platform is shown in Fig. 3b.

A laboratory-scale, continuous MPEF processing system consisting of a pulse power supply and a self-designed microchip was shown in Fig. 3(a,b), electrode spacing of the microchip was 100 µm, and the juice channel was set on top of the electrode. SSO were inoculated into sterilized blueberry juice, pulse width was 0.20 ms^51^. The influences of MPEF on the inactivation effect of SSO were studied by comparing the logarithmic decrease at different voltages (100–500 V). The logarithm value (lgS)52 was calculated as follows:

$$lg\,S=lg({N}_{0}/N),$$

where *N*_0_ is the number of microorganisms before treatment (CFU/mL), and *N* is the number of microorganisms after treatment (CFU/mL).

### Statistical analysis

All experiments were run in triplicate, and statistical analyses were performed using SPSS 16.0 statistical software and implemented using Origin 9.0 software. Anova and post-hoc test (Tukey; α = 0.05) were conducted to evaluate the inactivation effect of MPEF on SSO.

### Data Availability

The datasets generated during and/or analysed during the current study are available from the corresponding author on reasonable request.

## Electronic supplementary material

Dataset 1
