# Supplementary material for: Identification of spoilage microorganisms in blueberry juice and their inactivation by a microchip pulsed electric field system
Source: Sci Rep. 2018 May 25;8:8160. doi: 10.1038/s41598-018-26513-2 (PMC5970226; doi:10.1038/s41598-018-26513-2)
Supplement: Supplementary file 1 — Dataset 1 [file 41598_2018_26513_MOESM1_ESM.docx]

**Identification of spoilage microorganisms in blueberry juice and their inactivation by a microchip pulsed electric field system**

Ning Zhu^1^, Ning Yu^1^, Yue Zhu^1^, Yulong Wei^1^, Yanan Hou^1^, Haiping Zhang^1^,

Ai-dong Sun^1*^

*^1^Department of Food Science and Engineering, College of Biological Sciences and Technology,* *Beijing Forestry University, Beijing 100083, China*

**Corresponding author. Email:* [*adsun@bjfu.edu.cn*](mailto:adsun@bjfu.edu.cn)*; Telephone number: -008662336700； Fax number: -01062336700*

Supplementary Table S1 Comparison of the degree of spoiled blueberry juice in test tubes after 72h

| strain | Viscosity | Muddy | peculiar smell | Total colonies  (lg cfu/mL) | The degree of spoilage |
| --- | --- | --- | --- | --- | --- |
| 11 | + | - | + + | 3.67±0.06 | + |
| 12 | + + | + + | + + | 4.35±0.05 | + + |
| 13 | + + | + + | + + | 4.64±0.05 | + + |
| 14 | + + | + + + | + + + | 6.62±0.07 | + + + |
| 15 | + + | - | + | 3.41±0.04 | + |
| 16 | + + | + + | + + + | 5.86±0.06 | + + + |
| 18 | - | + + | + + + | 4.26±0.09 | + + |
| 19 | + | + + + | + + + | 6.89±0.06 | + + + |
| 21 | + + | + + | + + | 3.99±0.07 | + + |
| 23 | + + | + + | + + | 4.21±0.08 | + + |
| 24 | + + | - | + | 3.57±0.05 | + |
| 31 | + + | + + | + + | 4.52±0.09 | + + |
| 32 | + + | + | + + | 4.61±0.07 | + + |
| 34 | + + | + + | + + | 4.78±0.09 | + + |
| 35 | + | + + | + + | 4.31±0.08 | + + |
| 36 | + + | + + + | + + + | 6.72±0.07 | + + + |
| CK | - | - | - | <10 | - |

Notes: CK means control group; - means no viscosity, muddy, or peculiar smell; + means viscosity, muddy, or peculiar smell; a more number of + means higher intensity of muddy, precipitate, or acid, which means more serious degree of spoilage.

Supplementary Table S2 Original concentration of three kinds of microorganisms (lg cfu/mL)

| Strain | *Cryptococcus* | *Pichia* | *Meyerozyma* |
| --- | --- | --- | --- |
| *lgN_0_* | 5.93±0.11 | 5.86±0.09 | 6.08±0.10 |

Supplementary Table S3 Changes in quality parameters in fresh juice and treated by MPEF and HTST

| Blueberry  juices | Vitamin C (μg/mL) | Total phenols  (μg/mL) | Titratable acid (mg/mL) | Soluble reducing sugar (mg/mL) |
| --- | --- | --- | --- | --- |
| Fresh | 41.95±0.61 | 39.78±0.57 | 2.08±0.07 | 8.11±0.03 |
| MPEF | 41.25±0.53 | 39.75±0.56 | 2.08±0.06 | 8.30±0.10 |
| HTST | 34.20±0.75^*^ | 39.36±0.73 | 2.08±0.10 | 8.46±0.11 |

Note: * Significant difference between treatment (MPEF, HTST) groups and control (Fresh) group (p<0.05)


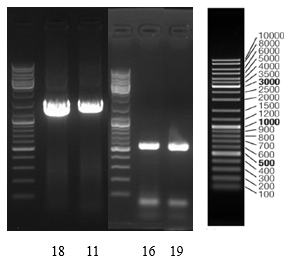


Supplementary Fig.S1a Electrophoresis results of PCR products 18, 11, 16, 19


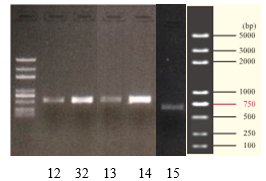


Supplementary Fig. S1b Electrophoresis results of PCR products 12, 32, 13, 14, 15


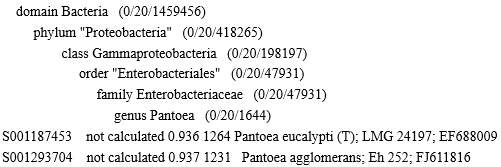


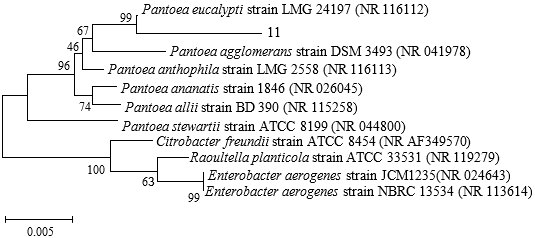


Supplementary Fig. S2a Sequence comparison results and phylogenetic tree of strain 11


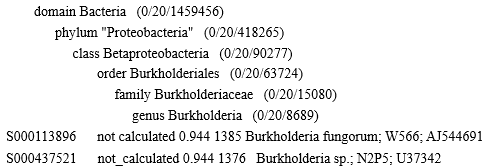


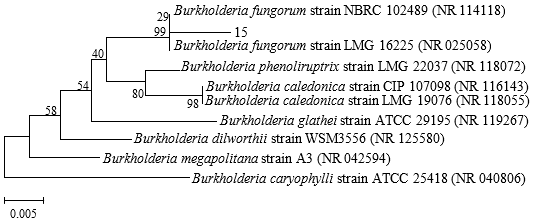


Supplementary Fig. S2b Sequence comparison results and phylogenetic tree of strain 15


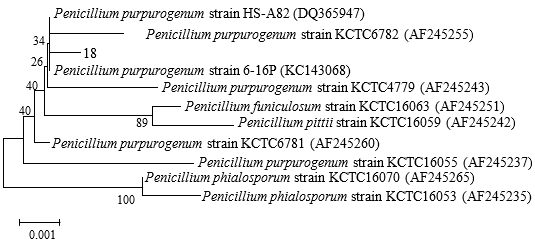


Supplementary Fig. S3 Phylogenetic tree of strain 18


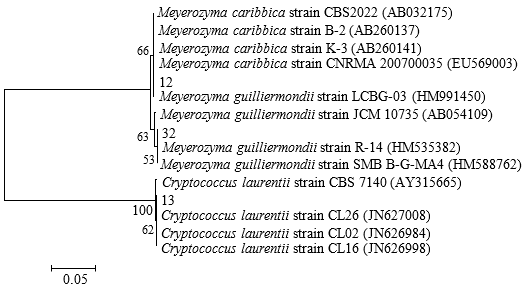


Supplementary Fig. S4 Phylogenetic tree of ITS sequences


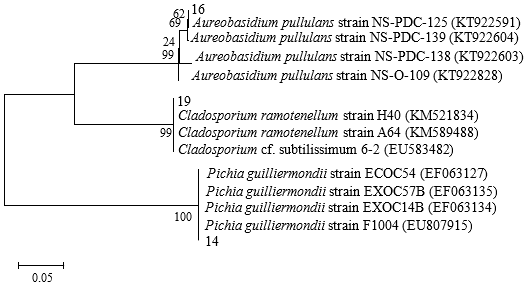


Supplementary Fig. S5 Phylogenetic tree of 26S rDNA sequences

Sequence data of strain 11 and phylogenetic tree

>11

GTCGGACGGTAGCACAGAGAGCTTGCTCTTGGGTGACGAGTGGGGGACGGGTGAGTAATGTCTGGGGATCTGCCCGATAGAGGGGGATAACCACTGGAAACGGTGGCTAATACCGCATAACGTCGCACGACCAAAGAGGGGGACCTTCGGGCCTCTCACTATCGGATGAACCCAGATGGGATTAGCTAGTAGGCGGGGTAATGGCCCACCTAGGCGACAATCCCTAGCTGGTCTGAGAGGATGACCAGCCACACTGGAACTGAGACACGGTCCAGACTCCTACGGGAGGCAGCAGTGGGGAATATTGCACAATGGGCGCAAGCCTGATGCACCCATGCCGCGTGTATGAAGAAAGCCTTCGGGTTGTAAAGTACTTTCAGCGGGGAGGAAGGCGATGTGGTTAATAACCCCGTCGATTGACGTTACCCGCACAAAAAACACCGGCTAACTCCGTGCCAGCAGCCGCGGTAATACGGAGGGTGCAAGCGTTAATCGGAATTACTGGGCGTAAAGCGCACGCACGCGGTCTGTTAAGTCAGATGTGAAATCCCCGGGCTTAACCTGGGAACTGCATTTGAAACTGGCAGGCTTGAGTCTTGTATAGGGGGGTAGAATTCCAGGTGTAGCGGTGAAATGCGTATAGATCTGGAGGAATACCGGTGGCGAAGGCGGCCCCCTGGACAAAGACTGACGCTCAGGTGCGAAAGCGTGGGGAGCAAACAGGATTAGATACCCTGGTAGTCCACGCCGTAAACGATGTCGACTTGGAGGTTGTTCCCTTGAGGAGTGGCTTCCGGAGCTAACGCGTTAAGTCGACCGCCTGGGGAGTACGGCCGCAAGGTTAAAACTCAAATGAATTGACGGGGGCCCGCACAAGCGGTGGAGCATGTGGTTTAATTCGATGCAACGCGAAGAACCTTACCTACTCTTGACATCCAGAGAACTTAGCAGAGATGGTTTGGTGCCTTCGGGAACTCTGAGACAGGTGCTGCATGGCTGTCGTCAGCTCGTGTTGTGAAATGTTGGGTTAAGTCCCGCAACGAGCGCAACCCTTATCCTTTGTTGCCAGCGCGTCATGGCGGGAACTCAAAGGAGACTGCCGGTGATAAACCGGAGGAAGGTGGGGATGACGTCAAGTCATCATGGCCCTTACGAGTAGGGCTACACACGTGCTACAATGGCGCATACAAAGAGAAGCGACCTCGCGAGAGCAAGCGGACCTCACAAAGTGCGTCGTAGTCCGGATCGGAGTCTGCAACTCGACTCCGTGAAGTCGGAATCGCTAGTAATCGTGGATCAGAATGCCACGGTGAATACGTTCCCGGGCCTTGTACACACCGCCCGTCACACCATGGGAGTGGGTTGCAAAAGAAGTAGGTAGCTTAACCTTCGGGAGG

>*Pantoea eucalypti* strain LMG 24197 (NR 116112)

GGACGGGTGAGTAATGTCTGGGGATCTGCCCGATAGAGGGGGATAACCACTGGAAACGGTGGCTAATACCGCATAACGTCGCAAGACCAAAGAGGGGGACCTTCGGGCCTCTCACTATCGGATGAACCCAGATGGGATTAGCTAGTAGGCGGGGTAATGGCCCACCTAGGCGACGATCCCTAGCTGGTCTGAGAGGATGACCAGCCACACTGGAACTGAGACACGGTCCAGACTCCTACGGGAGGCAGCAGTGGGGAATATTGCACAATGGGCGCAAGCCTGATGCAGCCATGCCGCGTGTATGAAGAAGGCCTTCGGGTTGTAAAGTACTTTCAGCGGGGAGGAAGGCGATGNGGTTAATAACCTCATCGATTGACGTTACCCGCAGAAGAAGCACCGGCTAACTCCGTGCCAGCAGCCGCGGTAATACGGAGGGTGCAAGCGTTAATCGGAATTACTGGGCGTAAAGCGCACGCAGGCGGTCTGTTAAGTCAGATGTGAAATCCCCGGGCTTAACCTGGGAACTGCATTTGAAACTGGCAGGCTTGAGTCTTGTAGAGGGGGGTAGAATTCCAGGTGTAGCGGTGAAATGCGTAGAGATCTGGAGGAATACCGGTGGCGAAGGCGGCCCCCTGGACAAAGACTGACGCTCAGGTGCGAAAGCGTGGGGAGCAAACAGGATTAGATACCCTGGTAGTCCACGCCGTAAACGATGTCGACTTGGAGGTTGTTCCCTTGAGGAGTGGCTTCCGGAGCTAACGCGTTAAGTCGACCGCCTGGGGAGTACGGCCGCAAGGTTAAAACTCAAATGAATTGACGGGGGCCCGCACAAGCGGTGGAGCATGTGGTTTAATTCGATGCAACGCGAAGAACCTTACCTACTCTTGACATCCAGAGAACTTAGCAGAGATGCTTTGGTGCCTTCGGGAACTCTGAGACAGGTGCTGCATGGCTGTCGTCAGCTCGTGTTGTGAAATGTTGGGTTAAGTCCCGCAACGAGCGCAACCCTTATCCTTTGTTGCCAGCGCGTGATGGCGGGAACTCAAAGGAGACTGCCGGTGATAAACCGGAGGAAGGTGGGGATGACGTCAAGTCATCATGGCCCTTACGAGTAGGGCTACACACGTGCTACAATGGCGCATACAAAGAGAAGCGACCTCGCGAGAGCAAGCGGACCTCACAAAGTGCGTCGTAGTCCGGATCGGAGTCTGCAACTCGACTCCGTGAAGTCGGAATCGCTAGTAATCGTGGATCAGAATGCCACGGTGAATACGTTCCCGGGCCTTGTACACACCGCCCGTCACACCATGGGAGTGGGTTGCAAAAGAAGTAGGTAGCTTAACCTTCG

> *Pantoea agglomerans* strain DSM 3493 (NR 041978)

GGCCTAACACATGCAAGTCTGACGGTAGCACAGAGGAGCTTGCTCCTTGGGTGACGAGTGGCGGACGGGTGAGTAATGTCTGGGGATCTGCCCGATAGAGGGGGATAACCACTGGAAACGGTGGCTAATACCGCATAACGTCGCAAGACCAAAGAGGGGGACCTTCGGGCCTCTCACTATCGGATGAACCCAGATGGGATTAGCTAGTAGGCGGGGTAATGGCCCACCTAGGCGACGATCCCTAGCTGGTCTGAGAGGATGACCAGCCACACTGGAACTGAGACACGGTCCAGACTCCTACGGGAGGCAGCAGTGGGGAATATTGCACAATGGGCGCAAGCCTGATGCAGCCATGCCGCGTGTATGAAGAAGGCCTTCGGGTTGTAAAGTACTTTCAGCGGGGAGGAAGGCGATGGGGTTAATAACCTTGTCGATTGACGTTACCCGCAGAAGAAGCACCGGCTAACTCCGTGCCAGCAGCCGCGGTAATACGGAGGGTGCAAGCGTTAATCGGAATTACTGGGCGTAAAGCGCACGCAGGCGGTCTGTTAAGTCAGATGTGAAATCCCCGGGCTTAACCTGGGAACTGCATTTGAAACTGGCAGGCTTGAGTCTTGTAGAGGGGGGTAGAATTCCAGGTGTAGCGGTGAAATGCGTAGAGATCTGGAGGAATACCGGTGGCGAAGGCGGCCCCCTGGACAAAGACTGACGCTCAGGTGCGAAAGCGTGGGGAGCAAACAGGATTAGATACCCTGGTAGTCCACGCCGTAAACGATGTCGACTTGGAGGTTGTTCCCTTGAGGAGTGGCTTCCGGAGCTAACGCGTTAAGTCGACCGCCTGGGGAGTACGGCCGCAAGGTTAAAACTCAAATGAATTGACGGGGGCCCGCACAAGCGGTGGAGCATGTGGTTTAATTCGATGCAACGCGAAGAACCTTACCTACTCTTGACATCCACGGAATTTGGCAGAGATGCCTTAGTGCCTTCGGGAACCGTGAGACAGGTGCTGCATGGCTGTCGTCAGCTCGTGTTGTGAAATGTTGGGTTAAGTCCCGCAACGAGCGCAACCCTTATCCTTTGTTGCCAGCGATTCGGTCGGGAACTCAAAGGAGACTGCCGGTGATAAACCGGAGGAAGGTGGGGATGACGTCAAGTCATCATGGCCCTTACGAGTAGGGCTACACACGTGCTACAATGGCGCATACAAAGAGAAGCGACCTCGCGAGAGCAAGCGGACCTCACAAAGTGCGTCGTAGTCCGGATCGGAGTCTGCAACTCGACTCCGTGAAGTCGGAATCGCTAGTAATCGTGGATCAGAATGCCACGGTGAATACGTTCCCGGGCCTTGTACACACCGCCCGTCACACCATGGGAGTGGGTTGCAAAAGAAGTAGGTAGCTTAACCTTCGGGAGGGCGCTTACCACTTTGTGATTCATGACTGGGGTGAAGTCGTAACAAGGTAACCGTAGGGG

>*Pantoea anthophila* strain LMG 2558 (NR 116113)

GGACGGGTGAGTAATGTCTGGGGATCTGCCCGATAGAGGGGGATAACCACTGGAAACGGTGGCTAATACCGCATAACGTCGCAAGACCAAAGAGGGGGACCTTCGGGCCTCTCACTATCGGATGAACCCAGATGGGATTAGCTAGTAGGCGGGGTAATGGCCCACCTAGGCGACGATCCCTAGCTGGTCTGAGAGGATGACCAGCCACACTGGAACTGAGACACGGTCCAGACTCCTACGGGAGGCAGCAGTGGGGAATATTGCACAATGGGCGCAAGCCTGATGCAGCCATGCCGCGTGTATGAAGAAGGCCTTCGGGTTGTAAAGTACTTTCAGCGGGGAGGAAGGCGGTGAGGTTAATAACCTYGCCGATTGACGTTACCCGCAGAAGAAGCACCGGCTAACTCCGTGCCAGCAGCCGCGGTAATACGGAGGGTGCAAGCGTTAATCGGAATTACTGGGCGTAAAGCGCACGCAGGCGGTCTGTTAAGTCAGATGTGAAATCCCCGGGCTTAACCTGGGAACTGCATTTGAAACTGGCAGGCTTGAGTCTYGTAGAGGGGGGTAGAATTCCAGGTGTAGCGGTGAAATGCGTAGAGATCTGGAGGAATACCGGTGGCGAAGGCGGCCCCCTGGACGAAGACTGACGCTCAGGTGCGAAAGCGTGGGGAGCAAACAGGATTAGATACCCTGGTAGTCCACGCCGTAAACGATGTCGACTTGGAGGTTGTTCCCTTGAGGAGTGGCTTCCGGAGCTAACGCGTTAAGTCGACCGCCTGGGGAGTACGGCCGCAAGGTTAAAACTCAAATGAATTGACGGGGGCCCGCACAAGCGGTGGAGCATGTGGTTTAATTCGATGCAACGCGAAGAACCTTACCTACTCTTGACATCCAGAGAACTTAGCAGAGATGCTTTGGTGCCTTCGGGAACTCTGAGACAGGTGCTGCATGGCTGTCGTCAGCTCGTGTTGTGAAATGTTGGGTTAAGTCCCGCAACGAGCGCAACCCTTATCCTTTGTTGCCAGCGATTCGGTCGGGAACTCAAAGGAGACTGCCGGTGATAAACCGGAGGAAGGTGGGGATGACGTCAAGTCATCATGGCCCTTACGAGTAGGGCTACACACGTGCTACAATGGCGCATACAAAGAGAAGCGACCTCGCGAGAGCAAGCGGACCTCACAAAGTGCGTCGTAGTCCGGATCGGAGTCTGCAACTCGACTCCGTGAAGTCGGAATCGCTAGTAATCGTGGATCAGAATGCCACGGTGAATACGTTCCCGGGCCTTGTACACACCGCCCGTCACACCATGGGAGTGGGTTGCAAAAGAAGTAGGTAGCTTAACC

>*Pantoea ananatis* strain 1846 (NR 026045)

AACACATGCAAGTCGGACGGTAGCACAGAGAGCTTGCTCTCGGGTGACGAGTGGCGGACGGGTGAGTAATGTCTGGGGATCTGCCCGATAGAGGGGGATAACCACTGGAAACGGTGGCTAATACCGCATAACGTCGCAAGACCAAAGAGGGGGACCTTCGGGCCTCTCACTATCGGATGAACCCAGATGGGATTAGCTAGTAGGCGGGGTAACGGCCCACCTAGGCGACGATCCCTAGCTGGTCTGAGAGGATGACCAGCCACACTGGAACTGAGACACGGTCCAGACTCCTACGGGAGGCAGCAGTGGGGAATATTGCACAATGGGCGCAAGCCTGATGCAGCCATGCCGCGTGTATGAAGAAGGCCTTCGGGTTGTAAAGTACTTTCAGCGGGGAGGAAGGCGATGTGGTTAATAACCGCATTGATTGACGTTACCCGCAGAAGAAGCACCGGCTAACTCCGTGCCAGCAGCCGCGGTAATACGGAGGGTGCAAGCGTTAATCGGAATTACTGGGCGTAAAGCGCACGCAGGCGGTCTGTTAAGTCAGATGTGAAATCCCCGGGCTTAACCTGGGAACTGCATTTGAAACTGGCAGGCTTGAGTCTCGTAGAGGGGGGTAGAATTCCAGGTGTAGCGGTGAAATGCGTAGAGATCTGGAGGAATACCGGTGGCGAAGGCGGCCCCCTGGACGAAGACTGACGCTCAGGTGCGAAAGCGTGGGGAGCAAACAGGATTAGATACCCTGGTAGTCCACGCCGTAAACGATGTCGACTTGGAGGTTGTTCCCTTGAGGAGTGGCTTCCGGAGCTAACGCGTTAAGTCGACCGCCTGGGGAGTACGGCCGCAAGGTTAAAACTCAAATGAATTGACGGGGGCCCGCACAAGCGGTGGAGCATATGGTTTAATTCGATGCAACGCGAAGAACCTTACCTACTCTTGACATCCAGAGAACTTAGCAGAGATGCTTTGGTGCCTTCGGGAACTCTGAGACAGGTGCTGCATGGCTGTCGTCAGCTCGTGTTGTGAAATGTTGGGTTAAGTCCCGCAACGAGCGCAACCCTTATCCTTTGTTGCCAGCGATTCGGTCGGGAACTCAAAGGAGACTGCCGGTGATAAACCGGAGGAAGGTGGGGATGACGTCAAGTCATCATGGCCCTTACGAGTAGGGCTACACACGTGCTACAATGGCGCATACAAAGAGAAGCGACCTCGCGAGAGCAAGCGGACCTCATAAAGTGCGTCGTAGTCCGGATCGGAGTCTGCAACTCGACTCCGTGAAGTCGGAATCGCTAGTAATCGTGGATCAGAATGCCACGGTGAATACGTTCCCGGGCCTTGTACACACCGCCCGTCACACCATGGGAGTGGGTTGCAAAAGAAGTAGGTAGCTTAACCTTCGGGAGGGCGCTTACCACTTTGTGATTCATGACTGGGGTGAAGTCGTAACAAGGT

>*Pantoea allii* strain BD 390 (NR 115258)

AGAGTTTGATCCTGGCTCAGATTGAACGCTGGCGGCAGGCCTAACACATGCAAGTCGGACGGTAGCACAGAGAGCTTGCTCTCGGGTGACGAGTGGCGGACGGGTGAGTAATGTCTGGGGATCTGCCCGATAGAGGGGGATAACCACTGGAAACGGTGGCTAATACCGCATAACGTCGCAAGACCAAAGAGGGGGACCTTCGGGCCTCTCACTATCGGATGAACCCAGATGGGATTAGCTAGTAGGCGGGGTAACGGCCCACCTAGGCGACGATCCCTAGCTGGTCTGAGAGGATGACCAGCCACACTGGAACTGAGACACGGTCCAGACTCCTACGGGAGGCAGCAGTGGGGAATATTGCACAATGGGCGCAAGCCTGATGCAGCCATGCCGCGTGTATGAAGAAGGCCTTCGGGTTGTAAAGTACTTTCAGCGGGGAGGAAGGCATTGTGGTTAATAACCGCAGTGATTGACGTTACCCGCAGAAGAAGCACCGGCTAACTCCGTGCCAGCAGCCGCGGTAATACGGAGGGTGCAAGCGTTAATCGGAATTACTGGGCGTAAAGCGCACGCAGGCGGTCTGTTAAGTCAGATGTGAAATCCCCGGGCTTAACCTGGGAACTGCATTTGAAACTGGCAGGCTTGAGTCTCGTAGAGGGGGGTAGAATTCCAGGTGTAGCGGTGAAATGCGTAGAGATCTGGAGGAATACCGGTGGCGAAGGCGGCCCCCTGGACGAAGACTGACGCTCAGGTGCGAAAGCGTGGGGAGCAAACAGGATTAGATACCCTGGTAGTCCACGCCGTAAACGATGTCGACTTGGAGGTTGTTCCCTTGAGGAGTGGCTTCCGGAGCTAACGCGTTAAGTCGACCGCCTGGGGAGTACGGCCGCAAGGTTAAAACTCAAATGAATTGACGGGGGCCCGCACAAGCGGTGGAGCATGTGGTTTAATTCGATGCAACGCGAAGAACCTTACCTACTCTTGACATCCAGAGAACTTAGCAGAGATGCTTTGGTGCCTTCGGGAACTCTGAGACAGGTGCTGCATGGCTGTCGTCAGCTCGTGTTGTGAAATGTTGGGTTAAGTCCCGCAACGAGCGCAACCCTTATCCTTTGTTGCCAGCGATTCGGTCGGGAACTCAAAGGAGACTGCCGGTGATAAACCGGAGGAAGGTGGGGATGACGTCAAGTCATCATGGCCCTTACGAGTAGGGCTACACACGTGCTACAATGGCGCATACAAAGAGAAGCGACCTCGCGAGAGCAAGCGGACCTCACAAAGTGCGTCGTAGTCCGGATCGGAGTCTGCAACTCGACTCCGTGAAGTCGGAATCGCTAGTAATCTGTGGATCAGAATGCCACGGTGAATACGTTCCCGGGCCTTGTACACACCGCCCGTCACACCATGGGAGTGGGTTGCAAAAGAAGTAGGTAGCTTAACCTCCGGGAGGGCGCTTACCACTTTGTGATTCATGACTGGGGTGAAGTCGTAACAAGGTAACCGTAGGGGAACCTGCGGCTGGATCACCTCCTT

>*Pantoea stewartii* strain ATCC 8199 (NR 044800)

AACACATGCAAGTCGGACGGTAGCACAGAGGAGCTTGCTCTCGGGTGACGAGTGGCGGACGGGTGAGTAATGTCTGGGAAACTGCCCGATGGAGGGGGATAACTACTGGAAACGGTAGCTAATACCGCATAACGTCGCAAGACCAAAGTGGGGGACCTCCGGGCCTCACACCATCGGATGTGCCCAGATGGGATTAGCTAGTAGGCGGGGTAACGGCCCACCTAGGCGACGATCCCTAGCTGGTCTGAGAGGATGACCAGCCACACTGGAACTGAGACACGGTCCAGACTCCTACGGGAGGCAGCAGTGGGGAATATTGCACAATGGGCGCAAGCCTGATGCAGCCATGCCGCGTGTATGAAGAAGGCCTTCGGGTTGTAAAGTACTTTCAGCGGGGAGGAAGGTGGTGAGGTTAATAACCTCATCAATTGACATTACCCGCAGAAGAAGCACCGGCTAACTCCGTGCCAGCAGCCGCGGTAATACGGAGGGTGCAAGCGTTAATCGGAATTACTGGGCGTAAAGCGCACGCAGGCGGTCTGTTAAGTCAGATGTGAAATCCCCGGGCTTAACCTGGGAACTGCATTTGAAACTGGCAGGCTTGAGTCTCGTAGAGGGGGGTAGAATTCCAGGTGTAGCGGTGAAATGCGTAGAGATCTGGAGGAATACCGGTGGCGAAGGCGGTCCCCTGGACGAAGACTGACGCTCAGGTGCGAAAGCGTGGGGAGCAAACAGGATTAGATACCCTGGTAGTCCACGCCGTAAACGATGTCGACTTGGAGGTTGTTCCCTTGAGGAGTGGCTTCCGGAGCTAACGCGTTAAGTCGACCGCCTGGGGAGTACGGCCGCAAGGTTAAAACTCAAATGAATTGACGGGGGCCCGCACAAGCGGTGGAGCATGTGGTTTAAT

TCGATGCAACGCGAAGAACCTTACCTACTCTTGACATCCAGCGAACTTGGCAGAGATGCCTTGGTGCCTTCGGGAACGCTGAGACAGGTGCTGCATGGCTGTCGTCAGCTCGTGTTGTGAAATGTTGGGTTAAGTCCCGCAACGAGCGCAACCCTTATCCTTTGTTGCCAGCGATTCGGTCGGGAACTCAAAGGAGACTGCCGGTGATAAACCGGAGGAAGGTGGGGATGACGTCAAGTCATCATGGCCCTTACGAGTAGGGCTACACACGTGCTACAATGGCGCATACAAAGAGAAGCGACCTCGCGAGAGCAAGCGGACCTCATAAAGTGCGTCGTAGTCCGGATCGGAGTCTGCAACTCGACTCCGTGAAGTCGGAATCGCTAGTAATCGTGGATCAGAATGCCACGGTGAATACGTTCCCGGGCCTTGTACACACCGCCCGTCACACCATGGGAGTGGGTTGCAAAAGAAGTAGGTAGCTTAACCCTCGGGAGGGCGCTTACTACTTTGTGATTCATGACTGGGGTGAAGTCGTAACAAGGT

> *Citrobacter freundii* strain ATCC 8454 (NR AF349570) ATGATGAAAAAATCGATATGCTGCGCGCTGCTGCTGACAGCCTCTTTCTCCACGTTTGCTGCCGCAAAAACAGAACAACAAATTGCCGATATCGTTAACCGCACCATCACACCACTGATGCAGGAGCAGGCTATTCCGGGTATGGCCGTGGCGATTATCTACGAGGGGAAACCTTATTACTTTACCTGGGGTAAAGCCGATATCGCCAATAACCACCCAGTCACGCAGCAAACGCTGTTTGAGCTAGGGTCGGTCAGTAAGACGTTTAACGGCGTGTTGGGCGGCGACGCTATCGCCCGCGGCGAAATTAAGCTCAGCGATCCGGTCACGAAATACTGGCCAGAACTGACAGGCAAACAGTGGCGGGGTATCAGCCTGCTGCACTTAGCCACCTATACAGCGGGTGGCCTGCCGCTGCAGATCCCCGATGACGTTACGGATAAAGCCGAATTACTGCGCTTTTATCAAAACTGGCAACCACAATGGACTCCGGGCGCTAAGCGTCTTTACGCTAACTCCAGCATTGGTCTGTTTGGTGCGCTGGCGGTGAAATCTTCAGGTATGAGCTACGAAGAGGCAATGACCAGACGCGTCCTGCAACCATTAAAACTGGCGCATACCTGGATTACGGTTCCGCAAAGCGAACAAAAAAACTATGCCTGGGGCTATCTCGAAGGGAAGCCTGTGCACGTTTCTCCGGGACAACTTGACGCCGAAGCCTATGGCGTGAAATCCAGCGTTATCGATATGGCCCGCTGGGTTCAGGCCAACATGGACGCCAGCCACGTTCAGGAGAAAACGCTCCAGCAGGGCATTGAGCTTGCGCAGTCTCGCTACTGGCGTATTGGTGATATGTACCAGGGATTAGGCTGGGAGATGCTGAACTGGCCGCTGAAAGCTGATTCGATCATCAACGGCAGCGACAGCAAAGTGGCATTGGCAGCGCTTCCCGCCGTTGAGGTAAACCCGCCAGCACCTGCCGTGAAAGCCTCATGGGTGCATAAAACAGGATCCACAGGCGGATTTGGCAGCTACGTTGCCTTCGTTCCAGAAAAAAACCTTGGCATCGTAATGTTGGCAAACAAAAGCTACCCCAACCCGGCTCGCGTCGAGGCGGCCTGGCGCATTCTTGAAAAACTGCAATAA

> *Raoultella planticola* strain ATCC 33531 (NR 119279)

TGAACGCTGGCGGCAGGCCTAACACATGCAAGTCGAGCGGTAGCACAGAGAGCTTGCTCTCGGGTGACGAGCGGCGGACGGGTGAGTAATGTCTGGGAAACTGCCTGATGGAGGGGGATAACTACTGGAAACGGTAGCTAATACCGCATAACGTCGCAAGACCAAAGTGGGGGACCTTCGGGCCTCATGCCATCAGATGTGCCCAGATGGGATTAGCTAGTAGGTGGGGTAATGGCTCACCTAGGCGACGATCCCTAGCTGGTCTGAGAGGATGACCAGCCACACTGGAACTGAGACACGGTCCAGACTCCTACGGGAGGCAGCAGTGGGGAATATTGCACAATGGGCGCAAGCCTGATGCAGCCATGCCGCGTGTATGAAGAAGGCCTTCGGGTTGTAAAGTACTTTCAGCGAGGAGGAAGGCRTTAAGGTTAATAACYTTRGTGATTGACGTTACTCGCAGAAGAAGCACCGGCTAACTCCGTGCCAGCAGCCGCGGTAATACGGAGGGTGCAAGCGTTAATCGGAATTACTGGGCGTAAAGCGCACGCAGGCGGTTTGTTAAGTCAGATGTGAAATCCCCGGGCTCAACCTGGGAACTGCATTTGAAACTGGCAAGCTTGAGTCTTGTAGAGGGGGGTAGAATTCCAGGTGTAGCGGTGAAATGCGTAGAGATCTGGAGGAATACCGGTGGCGAAGGCGGCCCCCTGGACAAAGACTGACGCTCAGGTGCGAAAGCGTGGGGAGCAAACAGGATTAGATACCCTGGTAGTCCACGCTGTAAACGATGTCGACTTGGAGGTTGTTCCCTTGAGGAGTGGCTTCCGGAGCTAACGCGTTAAGTCGACCGCCTGGGGAGTACGGCCGCAAGGTTAAAACTCAAATGAATTGACGGGGGCCCGCACAAGCGGTGGAGCATGTGGTTTAATTCGATGCAACGCGAAGAACCTTACCTACTCTTGACATCCAGRGAACTTAGCAGAGATGCTTTGGTGCCTTCGGGAACTCTGAGACAGGTGCTGCATGGCTGTCGTCAGCTCGTGTTGTGAAATGTTGGGTTAAGTCCCGCAACGAGCGCAACCCTTATCCTTTGTTGCCAGCGGTCCGGCCGGGAACTCAAAGGAGACTGCCAGTGATAAACTGGAGGAAGGTGGGGATGACGTCAAGTCATCATGGCCCTTACGAGTAGGGCTACACACGTGCTACAATGGCATATACAAAGAGAAGCGACCTCGCGAGAGCAAGCGGACCTCATAAAGTATGTCGTAGTCCGGATTGGAGTCTGCAACTCGACTCCATGAAGTCGGAATCGCTAGTAATCGTAGATCAGAATGCTACGGTGAATACGTTCCCGGGCCTTGTACACACCGCCCGTCACACCATGGGAGTGGGTTGCAAAAGAAGTAGGTAGCTTAACCTTCGGGAGGGCGCTTACCACTTTGTGATTCATGA

> *Enterobacter aerogenes* strain JCM1235 (NR 024643)

ACGCTGGCGGCAGGCCTAACACATGCAAGTCGAGCGGTAGCACAGAGAGCTTGCTCTCGGGTGACGAGCGGCGGACGGGTGAGTAATGTCTGGGAAACTGCCTGATGGAGGGGGATAACTACTGGAAACGGTAGCTAATACCGCATAACGTCGCAAGACCAAAGTGGGGGACCTTCGGGCCTCATGCCATCAGATGTGCCCAGATGGGATTAGCTAGTAGGTGGGGTAATGGCTCACCTAGGCGACGATCCCTAGCTGGTCTGAGAGGATGACCAGCCACACTGGAACTGAGACACGGTCCAGACTCCTACGGGAGGCAGCAGTGGGGAATATTGCACAATGGGCGCAAGCCTGATGCAGCCATGCCGCGTGTATGAAGAAGGCCTTCGGGTTGTAAAGTACTTTCAGCGAGGAGGAAGGCGTTAAGGTTAATAACCTTGGCGATTGACGTTACTCGCAGAAGAAGCACCGGCTAACTCCGTGCCAGCAGCCGCGGTAATACGGAGGGTGCAAGCGTTAATCGGAATTACTGGGCGTAAAGCGCACGCAGGCGGTCTGTCAAGTCGGATGTGAAATCCCCGGGCTCAACCTGGGAACTGCATTCGAAACTGGCAGGCTAGAGTCTTGTAGAGGGGGGTAGAATTCCAGGTGTAGCGGTGAAATGCGTAGAGATCTGGAGGAATACCGGTGGCGAAGGCGGCCCCCTGGACAAAGACTGACGCTCAGGTGCGAAAGCGTGGGGAGCAAACAGGATTAGATACCCTGGTAGTCCACGCCGTAAACGATGTCGACTTGGAGGTTGTGCCCTTGAGGCGTGGCTTCCGGAGCTAACGCGTTAAGTCGACCGCCTGGGGAGTACGGCCGCAAGGTTAAAACTCAAATGAATTGACGGGGGCCCGCACAAGCGGTGGAGCATGTGGTTTAATTCGATGCAACGCGAAGAACCTTACCTACTCTTGACATCCAGAGAACTTAGCAGAGATGCTTTGGTGCCTTCGGGAACTCTGAGACAGGTGCTGCATGGCTGTCGTCAGCTCGTGTTGTGAAATGTTGGGTTAAGTCCCGCAACGAGCGCAACCCTTATCCTTTGTTGCCAGCGGTCCGGCCGGGAACTCAAAGGAGACTGCCAGTGATAAACTGGAGGAAGGTGGGGATGACGTCAAGTCATCATGGCCCTTACGAGTAGGGCTACACACGTGCTACAATGGCATATACAAAGAGAAGCGACCTCGCGAGAGCAAGCGGACCTCATAAAGTATGTCGTAGTCCGGATTGGAGTCTGCAACTCGACTCCATGAAGTCGGAATCGCTAGTAATCGTAGATCAGAATGCTACGGTGAATACGTTCCCGGGCCTTGTACACACCGCCCGTCACACCATGGGAGTGGGTTGCAAAAGAAGTAGGTAGCTTAACCTTCGGGAGGNCGCTTTACCACTT

> *Enterobacter aerogenes* strain NBRC 13534 (NR 113614)

ATTGAACGCTGGCGGCAGGCCTAACACATGCAAGTCGAGCGGTAGCACAGAGAGCTTGCTCTCGGGTGACGAGCGGCGGACGGGTGAGTAATGTCTGGGAAACTGCCTGATGGAGGGGGATAACTACTGGAAACGGTAGCTAATACCGCATAACGTCGCAAGACCAAAGTGGGGGACCTTCGGGCCTCATGCCATCAGATGTGCCCAGATGGGATTAGCTAGTAGGTGGGGTAATGGCTCACCTAGGCGACGATCCCTAGCTGGTCTGAGAGGATGACCAGCCACACTGGAACTGAGACACGGTCCAGACTCCTACGGGAGGCAGCAGTGGGGAATATTGCACAATGGGCGCAAGCCTGATGCAGCCATGCCGCGTGTATGAAGAAGGCCTTCGGGTTGTAAAGTACTTTCAGCGAGGAGGAAGGCGTTAAGGTTAATAACCTTGGCGATTGACGTTACTCGCAGAAGAAGCACCGGCTAACTCCGTGCCAGCAGCCGCGGTAATACGGAGGGTGCAAGCGTTAATCGGAATTACTGGGCGTAAAGCGCACGCAGGCGGTCTGTCAAGTCGGATGTGAAATCCCCGGGCTCAACCTGGGAACTGCATTCGAAACTGGCAGGCTAGAGTCTTGTAGAGGGGGGTAGAATTCCAGGTGTAGCGGTGAAATGCGTAGAGATCTGGAGGAATACCGGTGGCGAAGGCGGCCCCCTGGACAAAGACTGACGCTCAGGTGCGAAAGCGTGGGGAGCAAACAGGATTAGATACCCTGGTAGTCCACGCCGTAAACGATGTCGACTTGGAGGTTGTGCCCTTGAGGCGTGGCTTCCGGAGCTAACGCGTTAAGTCGACCGCCTGGGGAGTACGGCCGCAAGGTTAAAACTCAAATGAATTGACGGGGGCCCGCACAAGCGGTGGAGCATGTGGTTTAATTCGATGCAACGCGAAGAACCTTACCTACTCTTGACATCCAGAGAACTTAGCAGAGATGCTTTGGTGCCTTCGGGAACTCTGAGACAGGTGCTGCATGGCTGTCGTCAGCTCGTGTTGTGAAATGTTGGGTTAAGTCCCGCAACGAGCGCAACCCTTATCCTTTGTTGCCAGCGGTNCGGCCGGGAACTCAAAGGAGACTGCCAGTGATAAACTGGAGGAAGGTGGGGATGACGTCAAGTCATCATGGCCCTTACGAGTAGGGCTACACACGTGCTACAATGGCATATACAAAGAGAAGCGACCTCGCGAGAGCAAGCGGACCTCATAAAGTATGTCGTAGTCCGGATTGGAGTCTGCAACTCGACTCCATGAAGTCGGAATCGCTAGTAATCGTAGATCAGAATGCTACGGTGAATACGTTCCCGGGCCTTGTACACACCGCCCGTCACACCATGGGAGTGGGTTGCAAAAGAAGTAGGTAGCTTAACCTTCGGGAGGGCGCTTACCACTTTGTGATTCATGACTGGGGTGAAG

Sequence data of strain 15 and phylogenetic tree

>15

GGATGACTGACTAGATTGACGCTGGCGGCATGCCTTACACATGCAAGTCGAACGGCAGCACGGGGGCAACCCTGGTGGCGAGTGGCGAACGGGTGAGTAATACATCGGAACGTGTCCTGTAGTGGGGGATAGCCCGGCGAAAGCCGGATTAATACCGCATACGCTCTACGGAGGAAAGGGGGGGATCTTAGGACCTCCCGCTACAGGGGCGGCCGATGGCAGATTAGCTAGTTGGTGGGGTAAAGGCCTACCAAGGCGACGATCTGTAGCTGGTCTGAGAGGACGACCAGCCACACTGGGACTGAGACACGGCCCAGACTCCTACGGGAGGCAGCAGTGGGGAATTTTGGACAATGGGGGCAACCCTGATCCAGCAATGCCGCGTGTGTGAAGAAGGCCTTCGGGTTGTAAAGCACTTTTGTCCGGAAAGAAAACGCCGTGGTTAATACCCGTGGCGGATGACGGTACCGGAAGAATAAGCACCGGCTAACTACGTGCCAGCAGCCGCGGTAATACGTCGTGACTGGGAAACCCCCTGGCGA

>*Burkholderia fungorum* strain NBRC 102489 (NR 114118)

ATTGAACGCTGGCGGCATGCCTTACACATGCAAGTCGAACGGCAGCACGGGGGCAACCCTGGTGGCGAGTGGCGAACGGGTGAGTAATACATCGGAACGTGTCCTGTAGTGGGGGATAGCCCGGCGAAAGCCGGATTAATACCGCATACGCTCTACGGAGGAAAGGGGGGGATCTTAGGACCTCCCGCTACAGGGGCGGCCGATGGCAGATTAGCTAGTTGGTGGGGTAAAGGCCTACCAAGGCGACGATCTGTAGCTGGTCTGAGAGGACGACCAGCCACACTGGGACTGAGACACGGCCCAGACTCCTACGGGAGGCAGCAGTGGGGAATTTTGGACAATGGGGGCAACCCTGATCCAGCAATGCCGCGTGTGTGAAGAAGGCCTTCGGGTTGTAAAGCACTTTTGTCCGGAAAGAAAACGNCGTGGTTAATACCCGTGGCGGNTGACGGTACCGGAAGAATAAGCACCGGCTAACTACGTGCCAGCAGCCGCGGTAATACGTAGGGTGCAAGCGTTAATCGGAATTACTGGGCGTAAAGCGTGCGCAGGCGGTCCGCTAAGACAGATGTGAAATCCCCGGGCTTAACCTGGGAACTGCATTTGTGACTGGCGGGCTAGAGTATGGCAGAGGGGGGTAGAATTCCACGTGTAGCAGTGAAATGCGTAGAGATGTGGAGGAATACCGATGGCGAAGGCAGCCCCCTGGGCCAATACTGACGCTCATGCACGAAAGCGTGGGGAGCAAACAGGATTAGATACCCTGGTAGTCCACGCCCTAAACGATGTCAACTAGTTGTCGGGTCTTCATTGACTTGGTAACGTAGCTAACGCGTGAAGTTGACCGCCTGGGGAGTACGGTCGCAAGATTAAAACTCAAAGGAATTGACGGGGACCCGCACAAGCGGTGGATGATGTGGATTAATTCGATGCAACGCGAAAAACCTTACCTACCCTTGACATGTATGGAATCCTGCTGAGAGGTGGGAGTGCCCGAAAGGGAGCCATAACACAGGTGCTGCATGGCTGTCGTCAGCTCGTGTCGTGAGATGTTGGGTTAAGTCCCGCAACGAGCGCAACCCTTGTCCCTAGTTGCTACGCAAGAGCACTCTAGGGAGACTGCCGGTGACAAACCGGAGGAAGGTGGGGATGACGTCAAGTCCTCATGGCCCTTATGGGTAGGGCTTCACACGTCATACAATGGTCGGAACAGAGGGTCGCCAACCCGCGAGGGGGAGCCAATCCCAGAAAACCGATCGTAGTCCGGATCGCACTCTGCAACTCGAGTGCGTGAAGCTGGAATCGCTAGTAATCGCGGATCAGCATGCCGCGGTGAATACGTTCCCGGGTCTTGTACACACCGCCCGTCACACCATGGGAGTGGGTTTTACCAGAAGTGGCTAGTCTAACCGCAAGGAGGACGGTCACCACGGTAGGATTCATGACTGGGGTGAAG

>*Burkholderia fungorum* strain LMG 16225 (NR 025058)

GCGGCATGCCTTACACATGCAAGTCGAACGGCAGCACGGGGGCAACCCTGGTGGCGAGTGGCGAACGGGTGAGTAATACATCGGAACGTGTCCTGTAGTGGGGGATAGCCCGGCGAAAGCCGGATTAATACCGCATACGCTCTACGGAGGAAAGGGGGGGATCTTAGGACCTCCCGCTACAGGGGCGGCCGATGGCAGATTAGCTAGTTGGTGGGGTAAAGGCCTACCAAGGCGACGATCTGTAGCTGGTCTGAGAGGACGACCAGCCACACTGGGACTGAGACACGGCCCAGACTCCTACGGGAGGCAGCAGTGGGGAATTTTGGACAATGGGGGCAACCCTGATCCAGCAATGCCGCGTGTGTGAAGAAGGCCTTCGGGTTGTAAAGCACTTTTGTCCGGAAAGAAAACGCCGTGGTTAATACCCGTGGCGGATGACGGTACCGGAAGAATAAGCACCGGCTAACTACGTGCCAGCAGCCGCGGTAATACGTAGGGTGCAAGCGTTAATCGGAATTACTGGGCGTAAAGCGTGCGCAGGCGGTCCGCTAAGACAGATGTGAAATCCCCGGGCTTAACCTGGGAACTGCATTTGTGACTGGCGGGCTAGAGTATGGCAGAGGGGGGTAGAATTCCACGTGTAGCAGTGAAATGCGTAGAGATGTGGAGGAATACCGATGGCGAAGGCAGCCCCCTGGGCCAATACTGACGCTCATGCACGAAAGCGTGGGGAGCAAACAGGATTAGATACCCTGGTAGTCCACGCCCTAAACGATGTCAACTAGTTGTCGGGTCTTCATTGACTTGGTAACGTAGCTAACGCGTGAAGTTGACCGCCTGGGGAGTACGGTCGCAAGATTAAAACTCAAAGGAATTGACGGGGACCCGCACAAGCGGTGGATGATGTGGATTAATTCGATGCAACGCGAAAAACCTTACCTACCCTTGACATGTATGGAATCCTGCTGAGAGGTGGGAGTGCCCGAAAGGGAGCCATAACACAGGTGCTGCATGGCTGTCGTCAGCTCGTGTCGTGAGATGTTGGGTTAAGTCCCGCAACGAGCGCAACCCTTGTCCCTAGTTGCTACGCAAGAGCACTCTAGGGAGACTGCCGGTGACAAACCGGAGGAAGGTGGGGATGACGTCAAGTCCTCATGGCCCTTATGGGTAGGGCTTCACACGTCATACAATGGTCGGAACAGAGGGTCGCCAACCCGCGAGGGGGAGCCAATCCCAGAAAACCGATCGTAGTCCGGATCGCACTCTGCAACTCGAGTGCGTGAAGCTGGAATCGCTAGTAATCGCGGATCAGCATGCCGCGGTGAATACGTTCCCGGGTCTTGTACACACCGCCCGTCACACCATGGGAGTGGGTTTTACCAGAAGTGGCTAGTCTAACCGCAAGGAGGACGGTCACCACGGTAGGATTCATGACTGGGGTGAAGTCGTAACAAGGTAGCCGTATCGGAAGG

>*Burkholderia dilworthii* strain WSM3556 (NR 125580)

CCTGGCTCAGATTGAACGCTGGCGGCATGCCTTACACATGCAAGTCGAACGGCAGCACGGGGGCAACCCTGGTGGCGAGTGGCGAACGGGTGAGTAATACATCGGAACGTGTCCTGTAGTGGGGGATAGCCCGGCGAAAGCCGGATTAATACCGCATACGCTCTGCGGAGGAAAGCGGGGGATCTTAGGACCTCGCGCTACAGGGGCGGCCGATGGCAGATTAGCTAGTTGGTGGGGTAAAGGCCTACCAAGGCGACGATCTGTAGCTGGTCTGAGAGGACGACCAGCCACACTGGGACTGAGACACGGCCCAGACTCCTACGGGAGGCAGCAGTGGGGAATTTTGGACAATGGGGGCAACCCTGATCCAGCAATGCCGCGTGTGTGAAGAAGGCCTTCGGGTTGTAAAGCACTTTTGTCCGGAAAGAAAGCTCCCGGGTTAATACCCTGGGGGGATGACGGTACCGGAAGAATAAGCACCGGCTAACTACGTGCCAGCAGCCGCGGTAATACGTAGGGTGCAAGCGTTAATCGGAATTACTGGGCGTAAAGCGTGCGCAGGCGGTTCGCTAAGACAGATGTGAAATCCCCGGGCTTAACCTGGGAACTGCATTTGTGACTGGCGGGCTAGAGTATGGCAGAGGGGGGTAGAATTCCACGTGTAGCAGTGAAATGCGTAGAGATGTGGAGGAATACCGATGGCGAAGGCAGCCCCCTGGGCCAATACTGACGCTCATGCACGAAAGCGTGGGGAGCAAACAGGATTAGATACCCTGGTAGTCCACGCCCTAAACGATGTCAACTAGTTGTCGGGCCTTCATTGGCTTGGTAACGTAGCTAACGCGTGAAGTTGACCGCCTGGGGAGTACGGTCGCAAGATTAAAACTCAAAGGAATTGACGGGGACCCGC

ACAAGCGGTGGATGATGTGGATTAATTCGATGCAACGCGAAAAACCTTACCTACCCTTGACATGTATGGAACCTGGCTGAGAGGTCAGGGTGCCCGAAAGGGAGCCATAACACAGGTGCTGCATGGCTGTCGTCAGCTCGTGTCGTGAGATGTTGGGTTAAGTCCCGCAACGAGCGCAACCCTTGTCCCTAGTTGCTACGCAAGAGCACTCCAGGGAGACTGCCGGTGACAAACCGGAGGAAGGTGGGGATGACGTCAAGTCCTCATGGCCCTTATGGGTAGGGCTTCACACGTCATACAATGGTCGGAACAGAGGGTCGCCAACCCGCGAGGGGGAGCCAATCCCAGAAAACCGATCGTAGTCCGGATCGCACTCTGCAACTCGAGTGCGTGAAGCTGGAATCGCTAGTAATCGCGGATCAGCATGCCGCGGTGAATACGTTCCCGGGTCTTGTACACACCGCCCGTCACACCATGGGAGTGGGTTTCACCAGAAGTAGGTAGCCTAACCTGCAAAGGAGGGCGCTTACCACGGTGGGATTCATGACTGGGGTGAAGTCGTAACAAGGTAGCCGTATCGGAAGGTGCGGCTGGATC

>*Burkholderia phenoliruptrix* strain LMG 22037 (NR 118072)

AGTGGCGAACGGGTGAGTAATACATCGGAACGTGTCCTGTAGTGGGGGATAGCCCGGCGAAAGCCGGATTAATACCGCATACGCTCTGCGGAGGAAAGCGGGGGATCCTTCGGGACCTCGCGCTACAGGGGCGGCCGATGGCAGATTAGCTAGTTGGTGGGGTAAAGGCCTACCAAGGCGACGATCTGTAGCTGGTCTGAGAGGACGACCAGCCAAACTGGGACTGAGACAGGGCCCAGACTNNTACGGGAGGCAGCAGTGGGGAATTTTGGACAATGGGGGCAACCCTGATCCAGCAATGCCGCGTGTGTGAAGAAGGCCTTCGGGTTGTAAAGCACTTTTGTCCGGAAAGAAAACGTCGTGGCTAATACCCGTGGCGGATGACGGTACCGGAAGAATAAGCACCGGCTAACTACGTGCCAGCAGCCGCGGTAATACGTAGGGTGCAAGCGTTAATCGGAATTACTGGGCGTAAAGCGTGCGCAGGCGGTTCGCTAAGACAGATGTGAAATCCCCGGGCTTAACCTGGGAACTGCATTTGTGACTGGCGGGCTAGAGTATGGCAGAGGGGGGTAGAATTCCACGTGTAGCAGTGAAATGCGTAGAGATGTGGAGGAATACCGATGGCGAAGGCAGCCCCCTGGGCCAATACTGACGCTCATGCACGAAAGCGTGGGGAGCAAACAGGATTAGATACCCTGGTAGTCCACGCCCTAAACGATGTCAACTGGTTGTCGGGCCTTCATTGGCTTGGTAACGTAGCTAACGCGTGAAGTTGACCGCCTGGGGAGTACGGTCGCAAGATTAAAACTCAAAGGAATTGACGGGGACCCGCACAAGCGGTGGATGATGTGGATTAATTCGATGCAACGCGAAAAACCTTACCTACCCTTGACATGGATGGAATCCCGCTGAGAGGTGGGAGTGCCCGAAAGGGAGCCATCACACAGGTGCTGCATGGCTGTCGTCAGCTCGTGTCGTGAGATGTTGGGTTAAGTCCCGCAACGAGCGCAACCCTTGTCCCTAGTTGCTACGCAAGAGCACTCCAGGGAGACTGCCGGTGACAAACCGGAGGAAGGTGGGGATGACGTCAAGTCCTCATGGCCCTTATGGGTAGGGCTTCACA

>*Burkholderia caledonica* strain CIP 107098 (NR 116143)

GGCGAGTGGCGAACGGGTGAGTAATACATCGGAACGTGTCCTGTAGTGGGGGATAGCCCGGCGAAAGCCGGATTAATACCGCATACGCTCTGCGGAGGAAAGCGGGGGATCCTTCGGGACCTCGCGCTACAGGGGCGGCCGATGGCAGATTAGCTAGTTGGTGGGGTAAAGGCCTACCAAGGCGACGATCTGTAGCTGGTCTGAGAGGACGACCAGCCACACTGGGACTGAGACACGGCCCAGACTCCTACGGGAGGCAGCAGTGGGGAATTTTGGACAATGGGCGCAAGCCTGATCCAGCAATGCCGCGTGTGTGAAGAAGGCCTTCGGGTTGTAAAGCACTTTTGTCCGGAAAGAAAACCGTGGCTTAATACCCGTGGGGGATGACGGTACCGGAAGAATAAGCACCGGCTAACTACGTGCCAGCAGCCGCGGTAATACGTAGGGTGCAAGCGTTAATCGGAATTACTGGGCGTAAAGCGTGCGCAGGCGGTTCGCTAAGACAGATGTGAAATCCCCGGGCTTAACCTGGGAACTGCATTTGTGACTGGCGGGCTAGAGTATGGCAGAGGGGGGTAGAATTCCACGTGTAGCAGTGAAATGCGTAGAGATGTGGAGGAATACCGATGGCGAAGGCAGCCCCCTGGGCCAATACTGACGCTCATGCACGAAAGCGTGGGGAGCAAACAGGATTAGATACCCTGGTAGTCCACGCCCTAAACGATGTCAACTAGTTGTCGGGTCTTCATTGACTTGGTAACGTAGCTAACGCGTGAAGTTGACCGCCTGGGGAGTACGGTCGCAAGATTAAAACTCAAAGGAATTGACGGGGACCCGCACAAGCGGTGGATGATGTGGATTAATTCGATGCAACGCGAAAAACCTTACCTACCCTTGACATGTATGGAAT

CCTGCTGAGAGGTGGGAGTGCCCGAAAGGGAGCCATAACACAGGTGCTGCATGGCTGTCGTCAGCTCGTGTCGTGAGATGTTGGGTTAAGTCCCGCAACGAGCGCAACCCTTGTCCCTAGTTGCTACGCAAGAGCACTCCAGGGAGACTGCCGGTGACAAACCGGAGGAAGGTGGGGATGACGTCAAGTCCTCATGGCCCTTATGGGTAGGGCTTCACACGTCATACAATGGTCGGAACAGAGGGTCGCCAACCCGCGAGGGGGAGCCAATCCCAGAAAACCGATCGTAGTCCGGATCGCACTCTGCAACTCGAGTGCGTGAAGCTGGAATCGCTAGTAATCGCGGATCAGCATGCCGCGGTGAATACGTTCCCGGGTCTTGTACACACCGCCCGTCACACCATG

>*Burkholderia caledonica* strain LMG 19076 (NR 118055)

AGTGGCGAACGGGTGAGTAATACATCGGAACGTGTCCTGTAGTGGGGGATAGCCCGGCGAAAGCCGGATTAATACCGCATACGCTCTGCGGAGGAAAGCGGGGGATCCTTCGGGACCTCGCGCTACAGGGGCGGCCGATGGCAGATTAGCTAGTTGGTGGGGTAAAGGCCTACCAAGGCGACGATCTGTAGCTGGTCTGAGAGGACGACCAGCCACACTGGGACTGAGACACGGCCCAGACTCCTACGGGAGGCAGCAGTGGGGAATTTTGGACAATGGGCGCAAGCCTGATCCAGCAATGCCGCGTGTGTGAAGAAGGCCTTCGGGTTGTAAAGCACTTTTGTCCGGAAAGAAAACCTCGTGGTTAATACCCGTGGGGGATGACGGTACCGGAAGAATAAGCACCGGCTAACTACGTGCCAGCAGCCGCGGTAATACGTAGGGTGCAAGCGTTAATCGGAATTACTGGGCGTAAAGCGTGCGCAGGCGGTTCGCTAAGACAGATGTGAAATCCCCGGGCTTAACCTGGGAACTGCATTTGTGACTGGCGGGCTAGAGTATGGCAGAGGGGGGTAGAATTCCACGTGTAGCAGTGAAATGCGTAGAGATGTGGAGGAATACCGATGGCGAAGGCAGCCCCCTGGGCCAATACTGACGCTCATGCACGAAAGCGTGGGGAGCAAACAGGATTAGATACCCTGGTAGTCCACGCCCTAAACGATGTCAACTAGTTGTCGGGTCTTCATTGACTTGGTAACGTAGCTAACGCGTGAAGTTGACCGCCTGGGGAGTACGGTCGCAAGATTAAAACTCAAAGGAATTGACGGGGACCCGCACAAGCGGTGGATGATGTGGATTAATTCGATGCAACGCGAAAAACCTTACCTACCCTTGACATGTATGGAACCCTGCTGAGAGGTGGGGGTGCCCGAAAGGGAGCCATAACACAGGTGCTGCATGGCTGTCGTCAGCTCGTGTCGTGAGATGTTGGGTTAAGTCCCGCAACGAGCGCAACCCTTGTCCCTAGTTGCTACGCAAGAGCACTCCAGGGAGACTGCCGGTGACAAACCGGAGGAAGGTGGGGATGACGTCAAGTCCTCATGGCCCTTATGGGTAGGGCTTCACA

>*Burkholderia caryophylli* strain ATCC 25418 (NR 040806)

TTACACATGCAAGTCGAACGGCAGCACGGGGGCAACCCTGGTGGCGAGTGGCGAACGGGTGAGTAATACATCGGAACGTGTCCTGGAGTGGGGGATAGCCCGGCGAAAGCCGGATTAATACCGCATACGCTCGGGAGAGGAAAGCGGGGGACCTTCGGGCCTCGCGCTGAAGGGGCGGCCGATGGCGGATTAGCTAGTTGGTGGGGTAAAGGCCTACCAAGGCGACGATCCGTAGCTGGTCTGAGAGGACGACCAGCCACACTGGGACTGAGACACGGCCCAGACTCCTACGGGAGGCAGCAGTGGGGAATTTTGGACAATGGGGGCAACCCTGATCCAGCAATGCCGCGTGTGCGAAGAAGGCCTTCGGGTTGTAAAGCACTTTTGTCCGGAAAGAAATCCTGCCTGATAATACCGGGCGGGGATGACGGTACCGGAAGAATAAGCACCGGCTAACTACGTGCCAGCAGCCGCGGTAATACGTAGGGTGCGAGCGTTAATCGGAATTACTGGGCGTAAAGCGTGCGCAGGCGGTTCGCTAAGACCGATGTGAAATCCCCGGGCTTAACCTGGGAACTGCATTGGTGACTGGCGGGCTAGAGTATGGCAGAGGGGGGTAGAATTCCACGTGTAGCAGTGAAATGCGTAGAGATGTGGAGGAATACCGATGGCGAAGGCAGCCCCCTGGGCCAATACTGACGCTCATGCACGAAAGCGTGGGGAGCAAACAGGATTAGATACCCTGGTAGTCCACGCCCTAAACGATGTCAACTAGTTGTTGGGGATTCATTTCCTTAGTAACGAAGCTAACGCGTGAAGTTGACCGCCTGGGGAGTACGGTCGCAAGATTAAAACTCAAAGGAATTGACGGGGACCCGCACAAGCGGTGGATGATGTGGATTAATTCGATGCAACGCGAAAAACCTTACCTACCCTTGACATGGACGGAATCCCGCTGAGAGGTGGGAGTGCTCGAAAGAGAACCGTCGCACAGGTGCTGCATGGCTGTCGTCAGCTCGTGTCGTGAGATGTTGGGTTAAGTCCCGCAACGAGCGCAACCCTTGTCCTTAGTTGCTACGCAAGAGCACTCTAAGGAGACTGCCGGTGACAAACCGGAGGAAGGTGGGGATGACGTCAAGTCCTCATGGCCCTTATGGGTAGGGCTTCACACGTCATACAATGGTCGGAACAGAGGGTCGCCAACCCGCGAGGGGGAGCCAATCCCAGAAAACCGATCGTAGTCCGGATTGCACTCTGCAACTCGAGTGCATGAAGCTGGAATCGCTAGTAATCGCGGATCAGCATGCCGCGGTGAATACGTTCCCGGGTCTTGTACACACCGCCCGTCACACCATGGGAGTGGGTTTTACCAGAAGTGGCTAGTCTAACCGCAAGGAGGACGGTCACCACGGTAGGATTCATGACTGGGGTGAAGTCGTAACAAGGTA

>*Burkholderia megapolitana* strain A3 (NR 042594)

ATTGAACGCTGGCGGCATGCCTTACACATGCAAGTCGAACGGCAGCGCGGGAGCAATCCTGGCGGCGAGTGGCGAACGGGTGAGTAATACATCGGAACGTGTCCTGTAGTGGGGGATAGCCCGGCGAAAGCCGGATTAATACCGCATACGCTCTACGGAGGAAAGGGGGGGATCTTAGGACCTCTCGCTACAGGGGCGGCCGATGGCGGATTAGCTAGTTGGTGGGGTAAAGGCCTACCAAGGCGACGATCCGTAGCTGGTCTGAGAGGACGACCAGCCACACTGGGACTGAGACACGGCCCAGACTCCTACGGGAGGCAGCAGTGGGGAATTTTGGACAATGGGGGCAACCCTGATCCAGCAATGCCGCGTGTGTGAAGAAGGCCTTCGGGTTGTAAAGCACTTTTGTCCGGAAAGAAATCATCCTGGTTAATACCTGGGGTGGATGACGGTACCGGAAGAATAAGCACCGGCTAACTACGTGCCAGCAGCCGCGGTAATACGTAGGGTGCAAGCGTTAATCGGAATTACTGGGCGTAAAGCGTGCGCAGGCGGTTCGCTAAGACAGATGTGAAATCCCCGGGCTTAACCTGGGAACTGCATTTGTGACTGGCGGGCTAGAGTATGGCAGAGGGGGGTAGAATTCCACGTGTAGCAGTGAAATGCGTAGAGATGTGGAGGAATACCGATGGCGAAGGCAGCCCCCTGGGCCAATACTGACGCTCATGCACGAAAGCGTGGGGAGCAAACAGGATTAGATACCCTGGTAGTCCACGCCCTAAACGATGTCAACTAGTTGTCGGGTCTTCATTGACTTGGTAACGTAGCTAACGCGTGAAGTTGACCGCCTGGGGAGTACGGTCGCAAGATTAAAACTCAAAGGAATTGACGGGGACCCGCACAAGCGGTGGATGATGTGGATTAATTCGATGCAACGCGAAAAACCTTACCTACCCTTGACATGTACGGAATCCTGCTGAGAGGTGGGAGTGCCCGAAAGGGAGCCGTAACACAGGTGCTGCATGGCTGTCGTCAGCTCGTGTCGTGAGATGTTGGGTTAAGTCCCGCAACGAGCGCAACCCTTGTCCCTAGTTGCTACGCAAGAGCACTCCAGGGAGACTGCCGGTGACAAACCGGAGGAAGGTGGGGATGACGTCAAGTCCTCATGGCCCTTATGGGTAGGGCTTCACACGTCATACAATGGTCGGAACAGAGGGTCGCCAACCCGCAAGGGGGAGCCAATCCCAGAAAACCGATCGTAGTCCGGATCGCAGTCTGCAACTCGACTGCGTGAAGCTGGAATCGCTAGTAATCGCGGATCAGCATGCCGCGGTGAATACGTTCCCGGGTCTTGTACACACCGCCCGTCACACCATGGGAGTGGGTTTTACCAGAAGTGGCTAGTCTAACCGCAAGGAGGACGGTCACCACGGTAGGATTCATGACTGGGGTGAAGTCGTAACAAGGTAGCCGTATCGGAAGG

>*Burkholderia glathei* strain ATCC 29195 (NR 119267)

AGTTTGATCCTGGCTCAGATTGAACGCTGGCGGCATGCCTTACACATGCAAGTCGAACGGCAGCACGGGGGCAACCCTGGTGGCGAGTGGCGAACGGGTGAGTAATACATCGGAACGTGTCCTGTAGTGGGGGATAGCCGGCGAAAGCCGGATTAATACCGCATACGATCTACGGAAGAAAGCGGGGGATCTTCGGACCTCGCGCTATAGGGGCGGCCGATGGCAGATTAGCTAGTTGGTGGGGTAAAGGCCTACCAAGGCGACGATCTGTAGCTGGTCTGAGAGGACGACCAGCCACACTGGGACTGAGACACGGCCCAGACTCCTACGGGAGGCAGCAGTGGGGAATTTTGGACAATGGGGGAAACCCTGATCCAGCAATGCCGCGTGTGTGAAGAAGGCCTTCGGGTTGTAAAGCACTTTTGTCCGGAAAGAAAACTTCGGGGCTAATACCTCTGGAGGATGACGGTACCGGAAGAATAAGCACCGGCTAACTACGTGCCAGCAGCCGCGGTAATACGTAGGGTGCGAGCGTTAATCGGAATTACTGGGCGTAAAGCGTGCGCAGGCGGTCTGTTAAGACAGATGTGAAATCCCCGGGCTTAACCTGGGAACTGCATTTGTGACTGGCAGGCTAGAGTATGGCAGAGGGGGGTAGAATTCCACGTGTAGCAGTGAAATGCGTAGAGATGTGGAGGAATACCGATGGCGAAGGCAGCCCCCTGGGCCAATACTGACGCTCATGCACGAAAGCGTGGGGAGCAAACAGGATTAGATACCCTGGTAGTCCACGCCCTAAACGATGTCAACTAGTTGTTGGGGATTCATTTCCTTAGTAACGTACGTAACGCGTGAAGTTGACCGCCTGGGGAGTACGGTCGCAAGATTAAAACTCAAAGGAATTGACGGG

GACCCGCACAAGCGGTGGATGATGTGGATTAATTCGATGCAACGCGAAAAACCTTACCTACCCTTGACATGGTCGGAACCCTGGTGAGAGCTGGGGGTGCTCGAAAGAGAACCGACACACAGGTGCTGCATGGCTGTCGTCAGCTCGTGTCGTGAGATGTTGGGTTAAGTCCCGCAACGAGCGCAACCCTTGTCCTTAGTTCGTACGCAAGAGCACTCTAAGGAGACTGCCGGTGACAAACCGGAGGAAGGTGGGGATGACGTCAAGTCCTCATGGCCCTTATGGGTAGGGCTTCACACGTCATACAATGGTCGGAACAGAGGGTCGCTAAGCCGCGAGGTGGAGCCAATCCCAGAAAACCGATCGTAGTCCGGATCGTAGTCTGCAACTCGACTACGTGAAGCTGGAATCGCTAGTAATCGCGGATCAGCATGCCGCGGTGAATACGTTCCCGGGTCTTGTACACACCGCCCGTCACACCATGGGAGTGGGTTTTACCAGAAGTGGCTAGTCTAACCGCAA

Sequence data of strain 18 and phylogenetic tree

>18

AGTATAAGCACTCTTTTACTGTGAAACTGCGAATGGCTCATTAAATCAGTTATCAGTTTATTTGATAGTACCCTACTACATGGATACCTGTGGTAATTCTAGAGCTAATACATGCGCAAAACCCCGACTTCGGAAGGGGTGTATTTATTAGATAAAAAACCAATGCCCTTCGGGGCTCCTTGGTGATTCATAATAACTTCACGAATCGCATGGCCTTGCGCCGGCGATGGTTCATTCAAATTTCTGCCCTATCAACTTTCGATGGTAGGATAGTGGCCTACCATGGTGGCAACGGGTAACGGGGAATTAGGGTTCGATTCCGGAGAGGGAGCCTGAGAAACGGCTACCACATCCAAGGAAGGCAGCAGGCGCGCAAATTACCCAATCCCGATACGGGGAGGTAGTGACAATAAATACTGATACAGGGCTCTTTTGGGTCTTGTAATTGGAATGAGAACAATCTAAATCCCTTAACGAGGAACAATTGGAGGGCAAGTCTGGTGCCAGCAGCCGCGGTAATTCCAGCTCCAATAGCGTATATTAAAGTTGTTGCAGTTAAAAAGCTCGTAGTTGAACCTTGGGCCCGTCCTGCCGGTCCGCCTCACCGCGAGTACTGGTCCGGATGGGCCTTTCTTTCTGGGGAATCCCATGGCCTTCACTGGCTGTGGCGGGGAACCAGGACTTTTACTGTGAAAAAATTAGAGTGTTCAAAGCAGGCCTTTGCTCGGATACATTAGCATGGAATAATAGAATAGGACGTGCGGTTCTATTTTGTTGGTTTCTAGGACCGCCGTAATGATTAATAGGGATAGTCGGGGGCGTCAGTATTCAGCTGTCAGAGGTGAAATTCTTGGATTTGCTGAAGACTAACTACTGCGAAAGCATTCGCCAAGGATGTTTTCATTAATCAGGGAACGAAAGTTAGGGGATCGAAGACGATCAGATACCGTCGTAGTCTTAACCATAAACTATGCCGACTAGGGATCGGGCGGGGTTTCTATGATGACCCGCTCGGCACCTTACGAGAAATCAAAGTTTTTGGGTTCTGGGGGGAGTATGGTCGCAAGGCTGAAACTTAAAGAAATTGACGGAAGGGCACCACAAGGCGTGGAGCCTGCGGCTTAATTTGACTCAACACGGGGAAACTCACCAGGTCCAGACAAAATAAGGATTGACAGATTGAGAGCTCTTTCTTGATCTTTTGGATGGTGGTGCATGGCCGTTCTTAGTTGGTGGAGTGATTTGTCTGCTTAATTGCGATAACGAACGAGACCTCGGCCCTTAAATAGCCCGGTCCGCGTTTGCGGGCCGCTGGCTTCTTAGGGGGACTATCGCTCAGC

>*Penicillium purpurogenum* strain HS-A82 (DQ 365947)

AAGCCATGCATGTCTAAGTATAAGCACTCTTTTACTGTGAAACTGCGAATGGCTCATTAAATCAGTTATCGTTTATTTGATAGTACCCTACTACATGGATACCTGTGGTAATTCTAGAGCTAATACATGCGCAAAACCCCGACTTCGGAAGGGGTGTATTTATTAGATAAAAAACCAATGCCCTTCGGGGCTCCTTGGTGATTCATAATAACTTCACGAATCGCATGGCCTTGCGCCGGCGATGGTTCATTCAAATTTCTGCCCTATCAACTTTCGATGGTAGGATAGTGGCCTACCATGGTGGCAACGGGTAACGGGGAATTAGGGTTCGATTCCGGAGAGGGAGCCTGAGAAACGGCTACCACATCCAAGGAAGGCAGCAGGCGCGCAAATTACCCAATCCCGATACGGGGAGGTAGTGACAATAAATACTGATACAGGGCTCTTTTGGGTCTTGTAATTGGAATGAGAACAATCTAAATCCCTTAACGAGGAACAATTGGAGGGCAAGTCTGGTGCCAGCAGCCGCGGTAATTCCAGCTCCAATAGCGTATATTAAAGTTGTTGCAGTTAAAAAGCTCGTAGTTGAACCTTGGGCCCGTCCTGCCGGTCCGCCTCACCGCGAGTACTGGTCCGGATGGGCCTTTCTTTCTGGGGAATCCCATGGCCTTCACTGGCTGTGGCGGGGAACCAGGACTTTTACTGTGAAAAAATTAGAGTGTTCAAAGCAGGCCTTTGCTCGGATACATTAGCATGGAATAATAGAATAGGACGTGCGGTTCTATTTTGTTGGTTTCTAGGACCGCCGTAATGATTAATAGGGATAGTCGGGGGCGTCAGTATTCAGCTGTCAGAGGTGAAATTCTTGGATTTGCTGAAGACTAACTACTGCGAAAGCATTCGCCAAGGATGTTTTCATTAATCAGGGAACGAAAGTTAGGGGATCGAAGACGATCAGATACCGTCGTAGTCTTAACCAT

AAACTATGCCGACTAGGGATCGGGCGGGGTTTCTATGATGACCCGCTCGGCACCTTACGAGAAATCAAAGTTTTTGGGTTCTGGGGGGAGTATGGTCGCAAGGCTGAAACTTAAAGAAATTGACGGAAGGGCACCACAAGGCGTGGAGCCTGCGGCTTAATTTGACTCAACACGGGGAAACTCACCAGGTCCAGACAAAATAAGGATTGACAGATTGAGAGCTCTTTCTTGATCTTTTGGATGGTGGTGCATGGCCGTTCTTAGTTGGTGGAGTGATTTGTCTGCTTAATTGCGATAACGAACGAGACCTCGGCCCTTAAATAGCCCGGTCCGCGTTTGCGGGCCGCTGGCTTCTTAGGGGGACTATCGGCTCAAGCCGATGGAAGTACGTGGCAATAACAGGTCTGTGATGCCCTTAGATGTTCTGGGCCGCACGCGCGCTACACTGACAGGGCCAGCGAGTACATCACCTTGGCCGAGAGGTCTGGGTAATCTTGTTAAACCCTGTCGTGCTGGGGATAGAGCATTGCAATTATTGCTCTTCAACGAGGAATGCCTAGTAGGCACGAGTCATCAGCTCGTGCCGATTACGTCCCTGCCCTTTGTACACACCGCCCGTCGCTACTACCG

ATTGAATGGCTCAGTGAGGCCTTCGGACTGGCTCAGAGGGGTTGGCAACGACCGCTCAGAGCCGGAAAGTTGGTCAAACTTGGTCATTTAGAGGAAGTAAAA

>*Penicillium purpurogenum* strain KCTC6782 (AF 245255)

AACCTGGTTGATCCTGCCAGTAGTCATATGCTTGTCTCAAAGATTAAGCCATGCATGTCTAAGTATAAGCACTCTTTTACTGTGAAACTGCGAATGGCTCATTAAATCAGTTATCGTTTATTTGATAGTACCCTACTACATGGATACCTGTGGTAATTCTAGAGCTAATACATGCGCAAAACCCCGACTTCGGAAGGGGTGTATTTATTAGATAAAAAACCAATGCCCTTCGGGGCTCCTTGGTGATTCATAATAACTTCACGAATCGCATGGCCTTGCGCCGGCGATGGTTCATTCAAATTTCTGCCCTATCAACTTTCGATGGTAGGATAGTGGCCTACCATGGTGGCAACGGGTAACGGGGAATTAGGGTTCGATTCCGGAGAGGGAGCCTGAGAAACGGCTACCACATCCAAGGAAAGCAGCAGGCGCGCAAATTACCCAATCCCGATACGGGGAGGTAGTGACAATAAATACTGATACAGGGTCCTTTTGGGTCTTGTAATTGGAATGAGAACAATCTAAATCCCTTAACGAGGAACAATTGGAGGGCAAGTCTGGTGCCAGCAGCCGCGGTAATTCCAGCTCCAATAGCGTATATTAAAGTTGTTGCAGTTAAAAAGCTCGTAGTTGAACCTTGGGCCCGTCCTGCCGGTCCGCCTCACCGCGAGTACTGGTCCGGATGGGCCTTTCTTTCTGGGGAATCCCATGGCCTTCACTGGCTGTGGCGGGGAACCAGGACTTTTACTGTGAAAAAATTAGAGTGTTCAAAGCAGGCCTTTGCTCGGATACATTAGCATGGAATAATAGAATAGGACGTGCGGTTCTATTTTGTTGGTTTCTAGGACCGCCGTAATGATTAATAGGGATAGTCGGGGGCGTCAGTATTCAGCTGTCAGAGGTGAAATTCTTGGATTTGCTGAAGACTAACTACTGCGAAAGCATTCGCCAAGGATGTTTTCATTAATCAGGGAACGAAAGTTAGGGGATCGAAGACGATCAGATACCGTCGTAGTCTTAACCATAAACTATGCCGACTAGGGATCGGGCGGGGTTTCTATGATGACCCGCTCGGCACCTTACGAGAAATCAAAGTTTTTGGGTTCTGGGGGGAGTATGGTCGCAAGGCTGAAACTTAAAGAAATTGACGGAAGGGCACCACAAGGCGTGGACCTGCGGCTTAATTTGACTCAACACGGGGAAACTCACCAGGTCCAGACAAAATAAGGATTGACAGATTGAGAGCTCTTTCTTGATCTTTTGGATGGTGGTGCATGGCCGTTCTTAGTTGGTGGAGTGATTTGTCTGCTTAATTGCGATAACGAACGAAACCTCGGCCCTTAAATAGCCCGGTCCGCGTTTGCGGGCCGCTGGCTTCTTAGGGGGACTATCGGCTCAAGCCGATGGAAGTACGTGGCAATAACAGGTCTGTGATGCCCTTAGATGTTCTGGGCCGCACGCGCGCTACACTGACAGGGCCAGCGAGTACATCACCTTGGCCGAGAGGTCTGGGTAATCTTGTTAAACCCTGTCGTGCTGGGGATAGAGCATTGCAATTATTGCTCTTCAACGAGGAATGCCTAGTAGGCACGAGTCATCAGCTCGTGCCG

ATTACGTCCCTGCCCTTTGTACACACCGCCCGTCGCTACTACCGATTGAATGGCTCAGTGAGGCCTTCGGACTGGCTCAAAGGGGTTGGCAACGACCGCTCAGAGCCGGAAAGTTGGTCAAACTTGGTCATTTAGAGGAATAAAATCGTAACAAGGTTTCCGTAGGTGAACCTGCAGAAGGATCAAG

>*Penicillium purpurogenum* strain 6-16P (KC 143068)

AGCACTCTTTTACTGTGAAACTGCGAATGGCTCATTAAATCAGTTATCGTTTATTTGATAGTACCCTACTACATGGATACCTGTGGTAATTCTAGAGCTAATACATGCGCAAAACCCCGACTTCGGAAGGGGTGTATTTATTAGATAAAAAACCAATGCCCTTCGGGGCTCCTTGGTGATTCATAATAACTTCACGAATCGCATGGCCTTGCGCCGGCGATGGTTCATTCAAATTTCTGCCCTATCAACTTTCGATGGTAGGATAGTGGCCTACCATGGTGGCAACGGGTAACGGGGAATTAGGGTTCGATTCCGGAGAGGGAGCCTGAGAAACGGCTACCACATCCAAGGAAGGCAGCAGGCGCGCAAATTACCCAATCCCGATACGGGGAGGTAGTGACAATAAATACTGATACAGGGCTCTTTTGGGTCTTGTAATTGGAATGAGAACAATCTAAATCCCTTAACGAGGAACAATTGGAGGGCAAGTCTGGTGCCAGCAGCCGCGGTAATTCCAGCTCCAATAGCGTATATTAAAGTTGTTGCAGTTAAAAAGCTCGTAGTTGAACCTTGGGCCCGTCCTGCCGGTCCGCCTCACCGCGAGTACTGGTCCGGATGGGCCTTTCTTTCTGGGGAATCCCATGGCCTTCACTGGCTGTGGCGGGGAACCAGGACTTTTACTGTGAAAAAATTAGAGTGTTCAAAGCAGGCCTTTGCTCGGATACATTAGCATGGAATAATAGAATAGGACGTGCGGTTCTATTTTGTTGGTTTCTAGGACCGCCGTAATGATTAATAGGGATAGTCGGGGGCGTCAGTATTCAGCTGTCAGAGGTGAAATTCTTGGATTTGCTGAAGACTAACTACTGCGAAAGCATTCGCCAAGGATGTTTTCATTAATCAGGGAACGAAAGTTAGGGGATCGAAGACGATCAGATACCGTCGTAGTCTTAACCATAAACTATGCCGACTAGGGATCG

GGCGGGGTTTCTATGATGACCCGCTCGGCACCTTACGAGAAATCAAAGTTTTTGGGTTCTGGGGGGAGTATGGTCGCAAGGCTGAAACTTAAAGAAATTGACGGAAGGGCACCACAAGGCGTGGAGCCTGCGGCTTAATTTGACTCAACACGGGGAAACTCACCAGGTCCAGACAAAATAAGGATTGACAGATTGAGAGCTCTTTCTTGATCTTTTGGATGGTGGTGCATGGCCGTTCTTAGTTGGTGGAGTGATTTGTCTGCTTAATTGCGATAACGAACGAGACCTCGGCCCTTAAATAGCCCGGTCCGCGTTTGCGGGCCGCTGGCTTCTTAGGGGGACTATCGGCTCAAGCCGATGGAAGTACGTGGCAATAACAGGTCTGTGATGCCCTTAGATGTTCTGGGCCGCACGCGCGCTACACTGACAGGGCCAGCGAGTACATCACCTTGGCCGAGAGGTCTGGGTAATCTTGTTAAACCCTGTCGTGCTGGGGATAGAGCATTGCAATTATTGCTCTTCAACGAGGAATGCCTAGTAGGCACGAGTCATCAGCTCGTGCCGATTACGTCCCTGCCCTTTGTACACACCGCCCGTCGCTACTACCGATTGAATGGCTCAGTGAGGCCTTCGGACTGGCTCAGAGGGGTTGGCAACGACCGCTCAGAGCCGGAA

>*Penicillium purpurogenum* strain KCTC6781 (AF 245260)

AACCTGGTTGATCCTGCCAGTAGTCATATGCTTGTCTCAAAGATTAAGCCATGCATGTCTAAGTATAAGCAMTCTTAACTGTGAAACTGCGAATGGCTCATTAAATCAGTTATCGTTTATTTGATAGTACCCTACTACATGGATACCTGTGGTAATTCTAGAGCTAATACATGCGCAAAACCCCGACTTCGGAAGGGGTGTATTTATTAGATAAAAAACCAATGCCCTTCGGGGCTCCTTGGTGATTCATAATAACTTCACGAATCGCATGGCCTTGCGCCGGCGATGGTTCATTCAAATTTCTGCCCTATCAACTTTCGATGGTAGGATAGTGGCCTACCATGGTGGCAACGGGTAACGGGGAATTAGGGTTCGATTCCGGAGAGGGAGCCTGAGAAACGGCTACCACATCCAAGGAAGGCAGCAGGCGCGCAAATTACCCAATCCCGATACGGGGAGGTAGTGACAATAAATACTGATACAGGGCTCTTTTGGGTCTTGTAATTGGAATGAGAACAATCTAAATCCCTTAACGAGGAACAATTGGAGGGCAAGTCTGGTGCCAGCAGCCGCGGTAATTCCAGCTCCAATAGCGTATATTAAAGTTGTTGCAGTTAAAAAGCTCGTAGTTGAACCTTGGGCCCGTCCTGCCGGTCCGCCTCACCGCGAGTACTGGTCCGGATGGCCTTTCTTTCTGGGGAATCCCATGGCCTTCACTGGCTGTGGCGGGGAACCAGGACTTTTACTGTGAAAAAATTAGAGTGTTCAAAGCAGGCCTTTGCTCGGATACATTAGCATGGAATAATAGAATAGGACGTGCGGTTCTATTTTGTTGGTTTCTAGGACCGCCGTAATGATTAATAGGGATAGTCGGGGGCGTCAGTATTCAGCTGTCAGAGGTGAAATTCTTGGATTTGCTGAAGACTAACTACTGCGAAAGCATTCGCCAAGGATGTTTTCATTAATCAGGGAACGAAAGTTAGGGGATCGAAGACGATCAGATACCGTCGTAGTCTTAACCATAAACTATGCCGACTAGGGATCGGGCGGGGTTTCTATRATGACCCGCTCGGCACCTTACGAGAAATCAAAGTTTTTGGGTTCTGGGGGGAGTATGGTCGCAAGGCTGAAACTTAAAGAAATTGACGGAAGGGCACCACAAGGCGTGGAGCTGCGGCTTAATTTGACTCAACACGGGGAAACTCACCAGGTCCAGACAAAATAAGGATTGACAGATTGAGAGCTCTTTCTTGATCTTTTGGATGGTGGTGCATGGCCGTTCTTAGTTGGTGGAGTGATTTGTCTGCTTAATTGCGATAACGAACGAGACCTCGGCCCTTAAATAGCCCGGTCCGCGTTTGCGGGCCGCTGGCTTCTTAGGGGGACTATCGGCTCAAGCCGATGGAAGTACGTGGCAATAACAGGTCTGTGATGCCCTTAGATGTTCTGGGCCGCACGCGCGCTACACTGACAGGGCCAGCGAGTACATCACCTTGGCCGAGAGGTCTGGGTAATCTTGTTAAACCCTGTCGTGCTGGGGATAGAGCATTGCAATTATTGCTCTTCAACGAGGAATGCCTAGTAGGCACGAGTCATCAGCTCGTGCCGAT

TACGTCCCTGCCCTTTGTACACACCGCCCGTCGCTACTACCGATTGAATGGCTCAGTGAGGCCTTCGGATTGGCTCAGAGGGGTTGGCAACGACCCCCCAGAGCCGAAAACTTGGCCAACCTCGGTCATTTAGAGGAAGTAAAAGTCGTAACAAGGTTTCCGTAGGTGAACCTGCAGAAGGATCAAG

>*Penicillium funiculosum* strain KCTC16063 (AF 245251)

AACCTGGTTGATCCTGCCAGTAGTCATATGCTTGTCTCAAAGATTAAGCCATGCATGTCTAAGTATAAGCATCTTTTACTGTGAAACTGCGAATGGCTCATTAAATCAGTTATCGTTTATTTGATAGTACCCTACTACATGGATACCTGTGGTAATTCTAGAGCTAATACATGCGCAAAACCCCGACTTCGGGAAGGGGTGTATTTATTAGATAAAAAACCAATGCCCTTCGGGGCTCCTTGGTGATTCATAATAACTTCACGAATCGCATGGCCTTGCGCCGGCGATGGTTCATTCAAATTTCTGCCCTATCAACTTTCGATGGTAGGATAGTGGCCTACCATGGTGGCAACGGGTAACGGGGAATTAGGGTTCGATTCCGGAGAGGGAGCCTGAGAAACGGCTACCACATCCAAGGAAGGCAGCAGGCGCGCAAATTACCCAATCCCGATACGGGGAGGTAGTGACAATAAATACTGATACAGGGCTCTTTTGGGTCTTGTAATTGGAATGAGAACAATCTAAATCCCTTAACGAGGAACAATTGGAGGGCAAGTCTGGTGCCAGCAGCCGCGGTAATTCCAGCTCCAATAGCGTATATTAAAGTTGTTGCAGTTAAAAAGCTCGTAGTTGAACCTTGGGCCGTCCTGGCCGGTCCGCCTCACCGCGAGTACTGGTCCGRATGGGCCTTTCTTTCTGGGGAATCCCATGGCCTTCACTGGCTGTGGCGGGGAACCAGGACTTTTACTGTGAAAAAATTAGAGTGTTCAAAGCAGGCCTTTGCTCGGATACATTAGCATGGAATAATAGAATAGGACGTGCGGTTCTATTTTGTTGGTTTCTAGGACCGCCGTAATGATTAATAGGGATAGTCGGGGGCGTCAGTATTCAGCTGTCAGAGGTGAAATTCTTGGATTTGCTGAAGACTAACTACTGCGAAAGCATTCGCCAAGGATGTTTTCATTAATCAGGGAACGAAAGTTAGGGGATCGAAGACGATCAGATACCGTCGTAGTCTTAACCATAAACTATGCCGACTAGGGATCGGGCGGGGTTCTATGATGACCCGCTCGGCACCTTACGAGAAATCAAAGTTTTTGGGTTCTGGGGGGAGTATGGTCGCAAGGCTGAAACTTAAAGAAATTGACGGAAGGGCACCACAAGGCGTGGAGCCTGCGGCTTAATTTGACTCAACACGGGGAAACTCACCAGGTCCAGACAAAATAAGGATTGACAGATTGAGAGCTCTTTCTTGATCTTTTGGATGGTGGTGCATGGCCGTTCTTAGTTGGTGGAGTGATTTGTCTGCTTAATTGCGATAACGAACGAGACCTCGGCCCTTAAATAGCCCGGTCCGCGTTTGCGGGCCGCTGGCTTCTTAGGGGGACTATCGGCTCAAGCCGATGGAAGTACGTGGCAATAACAGGTCTGTGATGCCCTTAGATGTTCTGGGCCGCACGCGCGCTACACTGACAGGGCCAGCGAGTACATCACCTTGGCCGAGAGGTCTGGGTAATCTTGTTAAACCCTGTCGTGCTGGGGATAGAGCATTGCAATTATTGCTCTTCAACGAGGAATGCCTAGTAGGCACGAGTCATCAGCTCGTGCCG

ATTACGTCCCTGCCCTTTGTACACACCGCCCGTCGCTACTACCGATTGAATGGCTCAGTGAGGCCTTCGGACTGGCTCAGAGGGGTTGGCAACGACCGCTCAGAGCCGGAAAGTTGGTCAAACTTGGTCATTTAGAGGAAGTAAAATCGTAACAAGGTTTCCGTAGGTGAACCTGCAGAAGGATCAAG

>*Penicillium pittii* strain KCTC16059 (AF 245242)

AACCTGGTTGATCCTGCCAGTAGTCATATGCTTGTCTCAAAGATTAAGCCATGCATGTCTAAGTATAAGCAMTCTTTTACTGTGAAACTGCGAATGGCTCATTAAATCAGTTATCGTTTATTTGATAGTACCCTACTACATGGATACCTGTGGTAATTCTAGAGCTAATACATGCGCAAAACCCCGACTTCGGAAGGGGTGTATTTATTAGATAAAAAACCAATGCCCTTCGGGGCTCCTTGGTGATTCATAATAACTTCACGAATCGCATGGCCTTGCGCCGGCGATGGTTCATTCAAATTTCTGCCCTATCAACTTTCGATGGTAGGATAGTGGCCTACCATGGTGGCAACGGGTAACGGGGAATTAGGGTTCGATTCCGGAGAGGGAGCCTGAGAAACGGCTACCACATCCAAGGAAGGCAGCAGGCGCGCAAATTACCCAATCCCGATCCGGGGAGGTAGTGACAATAAATACTGATACAGGGCTCTTTTGGGTCTTGTAATTGGAATGAGTACAATCTAAATCCCTTAACGAGGAACAATTGGAGGGCAAGTCTGGTGCCAGCAGCCGCGGTAATTCCAGCTCCAATAGCGTATATTAAAGTTGTTGCAGTTAAAAAGCTCGTAGTTGAACCTTGGGCCGTCCTTGCCGGTCCGCCTCACCGCGAGTACTGGTCCGGATGGGCCTTTCCTTCTGGGGAATCCCATGGCCTTCACTGGCTGTGGCGGGGAACCAGGACTTTTACTGTGAAAAAATTAGAGTGTTCAAAGCAGGCCTTTGCTCGGATACATTAGCATGGAATAATAGAATAGGACGTGTGGTTCTATTTTGTTGGTTTCTAGGACCGCCGTAATGATTAATAGGGATAGTCGGGGGCGTCAGTATTCAGCTGTCAGAGGTGAAATTCTTGGATTTGCTGAAGACTAACTACTGCGAAAGCATTCGCCAAGGATGTTTTCATTAATCAGGGAACGAAAGTTAGGGGATCGAAGACGATCAGATACCGTCGTAGTCTTAACCATAAACTATGCCGACTAGGGATCGGGCGGGTTTCTATGATGACCCGCTCGGCACCTTACGAGAAATCAAAGTTTTTGGGTTCTGGGGGGAGTATGGTCGCAAGGCTGAAACTTAAAGAAATTGACGGAAGGGCACCACAAGGCGTGGAGCTGCGGCTTAATTTGACTCAACACGGGGAAACTCACCAGGTCCAGACAAAATAAGGATTGACAGATTGAGAGCTCTTTCTTGATCTTTTGGATGGTGGTGCATGGCCGTTCTTAGTTGGTGGAGTGATTTGTCTGCTTAATTGCGATAACGAACGAGACCTCGGCCCTTAAATAGCCCGGTCCGCGTTTGCGGGCCGCTGGCTTCTTAGGGGGACTATCGGCTCAAGCCGATGGAAGTACGTGGCAATAACAGGTCTGTGATGCCCTTAGATGTTCTGGGCCGCACGCGCGCTACACTGACAGGGCCAGCGAGTACATCACCTTGGCCGAAAGGTCTGGGTAATCTTGTTAAACCCTGTCGTGCTGGGGATAGAGCATTGCAATTATTGCTCTTCAACGAGGAATGCCTAGTAGGCACGAGTCATCAGCTCGTGCCGA

TTACGTCCCTGCCCTTTGTACACACCGCCCGTCGCTACTACCGATTGAATGGCTCAGTGAGGCCTTCGGACTGGCTCAGGGGGGTTGGCAACGACCGCCCAGAGCCGGAAAGTTGGTCAAACTTGGTCATTTAGAGGAAGTAAAAGTCGTAACAAGGTTTCCGTAGGTGAACCTGCAGAAGGATCAAG

>*Penicillium phialosporum* strain KCTC16070 (AF 245265)

AACCTGGTTGATCCTGCCAGTAGTCATATGCTTGTCTCAAAGATTAAGCCATGCATGTCTAAGTATAAGCAMTTTGTACTGTGAAACTGCGAATGGCTCATTAAATCAGTTATCGTTTATTTGATAGTACCCTACTACATGGATACCTGTGGTAATTCTAGAGCTAATACATGCGCAAAACCCCGACTTCGGAAGGGGTGTATTTATTAGATAAAAAACCAATGCCCTTCGGGGCTCCTTGGTGATTCATAATAACTTAACGAATCGCATGGCCTTGCGCCGGCGATGGTTCATTCAAATTTCTGCCCTATCAACTTTCGATGGTAGGATAGTGGCCTACCATGGTGGCAACGGGTAACGGGGAATTAGGGTTCGATTCCGGAGAGGGAGCCTGAGAAACGGCTACCACATCCAAGGAAGGCAGCAGGCGCGCAAATTACCCAATCCCGATTCGGGGAGGTAGTGACAATAAATACTGATATAGGGCTCTTTTGGGTCTTATAATTGGAATGAGTACAATCTAAATCCCTTAACGAGGAACAATTGGAGGGCAAGTCTGGTGCCAGCAGCCGCGGTAATTCCAGCTCCAATAGCGTATATTAAAGTTGTTGCAGTTAAAAAGCTCGTAGTTGAACCTTGGGCCCGTCCTGCCGGTCCGCCTCACCGCGAGTACTGGTCCGGATGGGCCTTTCTTTCTGGGGAATCCCATGGCCTTCACTGGCTGTGGCGGGGAACCAGGACTTTTACTGTGAAAAAATTAGAGTGTTCAAAGCAGGCCTTTGCTCGGATACATTAGCATGGAATAATAAAATAGGACGTGCGGTTCTATTTTGTTGGTTTCTAGGACCGCCGTAATGATTAATAGGGATAGTCGGGGGCGTCAGTATTCAGCTGTCAGAGGTGAAATTCTTGGATTTGCTGAAGACTAACTACTGCGAAAGCATTCGCCAAGGATGTTTTCATTAATCAGGGAACGAAAGTTAGGGGATCGAAGACGATCAGATACCGTCGTAGTCTTAACCATAAACTATGCCGACTAGGGATCGGGCGGTGTTTCTATGATGACCCGCTCGGCACCTTACGAGAAATCAAAGTTTTTGGGTTCTGGGGGGAGTATGGTCGCAAGGCTGAAACTTAAAGAAATTGACGGAAGGGCACCACAAGGCGTGGAGCTGCGGCTTAATTTGACTCAACACGGGGAAACTCACCAGGTCCAGACAAAATAAGGATTGACAGATTGAGAGCTCTTTCTTGATCTTTTGGATGGTGGTGCATGGCCGTTCTTAGTTGGTGGAGTGATTTGTCTGCTTAATTGCGATAACGAACGAGACCTCGGCCCTTAAATAGCCCGGTCCGCATTTGCGGGCCGCTGGCTTCTTAGGGGGACTATCGGCTCAAGCCGATGGAAGTACGTGGCAATAACAGGTCTGTGATGCCCTTAGATGTTCTGGGCCGCACGCGCGCTACACTGACAGGGCCAGCGAGTACATCACCTTGGCCGAGAGGTCCGGGTAATCTTGTTAAACCCTGTCGTGCTGGGGATAGAGCATTGCAATTATTGCTCTTCAACGAGGAATGCCTAGTAGGCACGAGTCATCAGCTCGTGCCGA

TTACGTCCCTGCCCTTTGTACACACCGCCCGTCGCTACTACCGATTGAATGGCTCAGTGAGGCCTTCGGACTGGCTCAGGGAGGTTGGCAACGACCGCCCAGAGCCGGAAAGTTGGTCAAACTCTGTCATTTAGAGGATAAAAGTCGTAACAAGGTTTCCGTAGGTGAACCTGCAGAAGGATCAAG

>*Penicillium phialosporum* strain KCTC16053 (AF 245235)

AACCTGGTTGATCCTGCCAGTAGTCATATGCTTGTCTCAAAGATTAAGCCATGCATGTCTAAGTATAAGCACTTTGTACTGTGAAACTGCGAATGGCTCATTAAATCAGTTATCGTTTATTTGATAGTACCCTACTACATGGATACCTGTGGTAATTCTAGAGCTAATACATGCGCAAAACCCCGACTTCGGAAGGGGTGTATTTATTAGATAAAAAACCAATGCCCTTCGGGGCTCCTTGGTGATTCATAATAACTTAACGAATCGCATGGCCTTGCGCCGGCGATGGTTCATTCAAATTTCTGCCCTATCAACTTTCGATGGTAGGATAGTGGCCTACCATGGTGGCAACGGGTAACGGGGAATTAGGGTTCGATTCCGGAGAGGGAGCCTGAGAAACGGCTACCACATCCAAGGAAGGCAGCAGGCGCGCAAATTACCCAATCCCGATTCGGGGAGGTAGTGACAATAAATACTGATATAGGGCTCTTTTGGGTCTTATAATTGGAATGAGTACAATCTAAATCCCTTAACGAGGAACAATTGGAGGGCAAGTCTGGTGCCAGCAGCCGCGGTAATTCCAGCTCCAATAGCGTATATTAAAGTTGTTGCAGTTAAAAAGCTCGTAGTTGAACCTTGGGCCCGTCCTGCCGGTCCGCCTCACCGCGAGTACTGGTCCGGATGGGCCTTTCTTTCTGGGGAATCCCATGGCCTTCACTGGCTGTGGCGGGGAACCAGGACTTTTACTGTGAAAAAATTAGAGTGTTCAAAGCAGGCCTTTGCTCGGATACATTAGCATGGAATAATAAAATAGGACGTGCGGTTCTATTTTGTTGGTTTCTAGGACCGCCGTAATGATTAATAGGGATAGTCGGGGGCGTCAGTATTCAGCTGTCAGAAGTGAAATTCTTGGATTTGCTGAAGACTAACTACTGCGAAAGCATTCGCCAAGGATGTTTTCATTAATCAGGGAACGAAAGTTAGGGGATCGAAGACGATCAGATACCGTCGTAGTCTTAACCATAAACTATGCCGACTAGGGATCGGGCGGTGTTTCTATGATGACCCGCTCGGCACCTTACGAGAAATCAAAGTTTTTGGGTTCTGGGGGGAGTATGGTCGCAAGGCTGAAACTTATTGAAATTGACGGAAGGGCACCACAAGGCGTGGAGCCTGCGGCTTAATTTGACTCAACACGGGGAAACTCACCAGGTCCAGACAAAATAAGGATTGACAGATTGAGAGCTCTTTCTTGATCTTTTGGATGGTGGTGCATGGCCGTTCTTAGTTGGTGGAGTGATTTGTCTGCTTAATTGCGATAACGAACGAGACCTCGGCCCTTAAATAGCCCGGTCCGCATTTGCGGGCCGCTGGCTTCTTAGGGGGACTATCGGCTCAAGCCGATGGAAGTACGTGGCAATAACAGGTCTGTGATGCCCTTAGATGTTCTGGGCCGCACGCGCGCTACACTGACAGGGCCAGCGAGTACATCACCTTGGCCGAGAGGTCCGGGTAATCTTGTTAAACCCTGTCGTGCTGGGGATAGAGCATTGCAATTATTGCTCTTCAACGAGGAATGCCTAGTAGGCACGAGTCATCAGCTCGTGCCG

ATTACGTCCCTGCCCTTTGTACACACCGCCCGTCGCTACTACCGATTGAATGGCTCAGTGAGGCCTTCGGACTGGCTCAGGAGGTTGGCAACGACCGCCCAGAGCCGGAAAGTTGGTCAAACTTGGTCATTTAGAGGAAGTAAAAGTCGTAACAAGGTTTCCGTAGGTGAACCTGCAGAAGGATCAAG

>*Penicillium purpurogenum* strain KCTC16055 (AF 245237)

AACCTGGTTGATCCTGCCAGTAGTCATATGCTTGTCTCAAAGATTAAGCCATGCATGTCTAAGTATAAGCACTCTTGTACCGTGAAACTGCGAATGGCTCATTAAATCAGTTATCGTTTATTTGATAGTACCCTACTACATGGATACCTGTGGTAATTCTAGAGCTAATACATGCGCAAAACCCCGACTTCGGGAAGGGGTGTATTTATTAGATAAAAAACCAATGCCCCTCGGGGCTCCTTGGTGATTCATAATAACTTCACGAATCGCATGGCCTTGCGCCGGCGATGGTTCATTCAAATTTCTGCCCTATCAACTTTCGATGGTAGGATAGTGGCCTACCATGGTGGCAACGGGTAACGGGGAATTAGGGTTCGATTCCGGAGAGGGAGCCTGAGAAACGGCTACCACATCCAAGGAAGGCAGCAGGCGCGCAAATTACCCAATCCCGATACGGGGAGGTAGTGACAATAAATACTGATACAGGGTCTTTTGGGTCTTGTAATTGGAATGAGAACAATTTAAATCCCTTAACGAGGAACAATTGGAGGGCAAGTCTGGTGCCAGCAGCCGCGGTAATTCCAGCTCCAATAGCGTATATTAAAGTTCTTGCAGTTAAAAAGCTCGTAGTTGAACCTTGGGCCCGTCCTGCCGGTCCGCCTCACCGCGAGTACTGGTCCGGATGGGCCTTTCTTTCTGGGGAATCCCATGGCCTTCACTGGCTGTGGCGGGGAACCAGGACTTTTACTGTGAAAAAATTAGAGTGTTCAAAGCAGGCCTTTGCTCGGATACATTAGCATGGAATAATAGAATAGGACGTGCGGTTCTATTTTGTTGGTTTCTAGGACCGCCGTAATGATTAATAGGGATAGTCGGGGGCGTCAGTATTCAGCTGTCAGAGGTGAAATTCTTGGATTTGCTGAAGACTTACTACTGCGAAAGCATTCGCCAAGGATGTTTTCATTAATCAGGGAACGAAAGTTAGGGGATCGAAGACGATCAGATACCGTCGTAGTCTTAACCATAAACTATGCCGACTAGGGATCGGGCGGGTTTCTATGATGACCCGCTCGGCACCTTACGAGAAATCAAAGTTTTTGGGTTCTGGGGGGAGTATGGTCGCAAGGCTGAAACTTAAAGAAATTGACGGAAGGGCACCACAAGGCGTGGAGCCTGCGGCTTAATTTGACTCAACACGGGGAAACTCACCAGGTCCAGACAAAATAAGGATTGACAGATTGAGAGCTCTTTCTTGATCTTTTGGATGGTGGTGCATGGCCGTTCTTAGTTGGTGGAGTGATTTGTCTGCTTAATTGCGATAACGAACGAGACCTCGGCCCTTAAATAGCCCAGTTCGCGTTTGCGGGCTGCTGGCTTCTTAGGGGGACTATCGGCTCAAGCCGATGGAAGTACGTGGCAATAACAGGTCTGTGATGCCCTTAGATGTTCTGGGCCGCACGCGCGCTACACTGACAGGGCCAGCGAGTACATCACCTTGGCCGAGAGGCCTGGGTAATCTTGTTAAACCCTGTCGTGCTGGGGATAGAGCATTGCAATTATTGCTCTTCAACGAGGAATGCCTAGTAGGCACGAGTCATCAGCTCGTGCCG

ATTACGTCCCTGCCCTTTGTACACACCGCCCGTCGCTACTACCGATTGAATGGCTCAGTGAGGCCTTCGGACTGGCTCAGGGGGGTTGGCAACGACCGCCCAGAGCCGGAAAGTTGGTCAAACTTGGTCATTTAGAGGAAGTAAAAGTCGTAACAAGGTTTCCGTAGGTGAACCTGCAGAAGGATCAAG

>*Penicillium purpurogenum* strain KCTC4779 (AF 245243)

AACCTGGTTGATCCTGCCAGTAGTCATATGCTTGTCTCAAAGATTAAGCCATGCATGTCTAAGTATAAGCACTCTTTTACTGTGAAACTGCGAATGGCTCATTAAATCAGTTATCGTTTATTTGATAGTACCCTACTACATGGATACCTGTGGTAATTCTAGAGCTAATACATGCGCAAAACCCCGACTTCGGAAGGGGTGTATTTATTAGATAAAAAACCAATGCCCTTCGGGGCTCCTTGGTGATTCATAATAACTTCACGAATCGCATGGCCTTGCGCCGGCGATGGTTCATTCAAATTTCTGCCCTATCAACTTTCGATGGTAGGATAGTGGCCTACCATGGTGGCAACGGGTAACGGGGAATTAGGGTTCGATTCCGGAGAAGGAGCCTGAGAAACGGCTACCACATCCAAGGAAGGCAGCAGGCGCGCAAATTACCCAATCCCGATACGGGGAGGTAGTGACAATAAATACTGATACAGGGTTCTTTTGGGTCTTGTAATTGGAATGAGAACAATCTAAATCCCTTAACGAGGAACAATTGGAGGGCAAGTCTGGTGCCAGCAGCCGCTGGTAATTCCAGCTCCAATAGCGTATATTAAAGTTGTTGCAGTTAAAAAGCTCGTAGTTGAACCTTGGGCCCGTCCTGCCGGTCCGCCTCACCGCGAGTACTGGTCCGGATGGGCCTTTCTTTCTGGGGAATCCCATGGCCTTCACTGGCTGTGGCGGGGAACCAGGACTTTTACTGTGAAAAAATTAGAGTGTTCAAAGCAGGCCTTTGCTCGGATACATTAGCATGGAATAATAGAATAGGACGTGCGGTTCTATTTTGTTGGTTTCTAGGACCGCCGTAATGATTAATAGGGATAGTCGGGGGCGTCAGTATTCAGCTGTCAGAGGTGAAATTCTTGGATTTGCTGAARACTAACTACTGCGAAAGCATTCGCCAAGGATGTTTTCATTAATCAGGGAACGAAAGTTAGGGGATCSAAGACGATCAGATACCGTCGTAGTCTTAACCATAAACTATGCCGATTAGGGATCGGGCGGGGTTTCTATGATGACCCGCTCGGCACCTTACGAGAAATCAAAGTTTTTGGGTTCTGGGGGGAGTATGGTCGCAAGGCTGAAACTTAAAGAAATTGACGGAAGGGCACCACAAGGCGTGGAGCCTGCGGCTTAATTTGACTCAACACGGGGAAACTCACCAGGTCCAGACAAAATAAGGATTGACAGATTGAGAGCTCTTTCTTGATCTTTTGGATGGTGGTGCATGGCCGTTCTTAGTTGGTGGAGTGATTTGTCTGCTTAATTGCGATAACGAACGAGACTTCGGCCCTTAAATAGCCCCGTCCGCGTTTGCGGGCCGCTGGTTCTTAGGGGGATATCGGCTCAAGCCGATGGAAGTAMSTGGCAATAACAGGTCTGTGATGCCCTTAGATGTTCTGGGCCGCACGCGCGCTACACTGACAGGGCCAGCGAGTACATCACCTTGGCCGAGAAGTCTGGGTAATCTTGTTAAACCCTGTCGTGCTGGGGTTAGAGCATTGCAATTATTGCTCTTCAACGAGGAATGCYTAGTAGGCACGAGTCATCAGCTCGTGCCG

ATTACGTCCCTGCCCTTTGTACACACCGCCCGTCGCTACTACCGATTGAATGGCTCAGTGAGGCCTTCGGACTGGCTCARAGGGGTTGGCAACGACCGCTCAGAGCCGGAAAGTTGGTCAAACTTGGTCATTTARAGGAAGTAAAAGTCGTAACAAGGTTTCCGTAGGTGAACCTGCAGAAGGATCAAG

Data for Phylogenetic tree of 26S rDNA sequences

>14

AAGCGGAGGAAAAGAAACCAACAGGGATTGCCTTAGTAGCGGCGAGTGaAGCGGCAAAAGCTCAAATTTGAAATCTGGCGCCTTCGGTGTCCGAGTTGTAATTTGAAGATTGTAACCTTGGGGTTGGCTCTTGTCTATGTTTCTTGGAACAGGACGTCACAGAGGGTGAGAATCCCGTGCGATGAGATGCCCAATTCTATGTAAGGTGCTTTCGAAGAGTCGAGTTGTTTGGGAATGCAGCTCTAAGTGGGTGGTAAATTCCATCTAAAGCTAAATATTGGCGAGAGACCGATAGCGAACAAGTACAGTGATGGAAAGATGAAAAGAACTTTGAAAAGAGAGTGAAAAAGTACGTGAAATTGTTGAAAGGGAAGGGTTTGAGATCAGACTCGATATTTTGTGAGCCTTGCCTTCGTGGCGGGGTGACCCGCAGCTTATCGGGCCAGCATCGGTTTGGGCGGTAGGATAATGGCGTAGGAATGTGACTTTACTTCGGTGAAGTGTTATAGCCTGCGTTGATGCTGCCTGCCTAGACCGAGGACTGCGATTATCAAGGATGCTGgCATAATGATCCCAAACCGCCCGTCTTGACCAACGGACCA

>*Pichia guilliermondii* strain ECOC54 (EF 063127)

GCATATCCAATAAGCGGAGGAAAAGAAACCAACAGGGATTGCCTTAGTAGCGGCGAGTGAAGCGGCAAAAGCTCAAATTTGAAATCTGGCGCCTTCGGTGTCCGAGTTGTAATTTGAAGATTGTAACCTTGGGGTTGGCTCTTGTCTATGTTTCTTGGAACAGGACGTCACAGAGGGTGAGAATCCCGTGCGATGAGATGCCCAATTCTATGTAAGGTGCTTTCGAAGAGTCGAGTTGTTTGGGAATGCAGCTCTAAGTGGGTGGTAAATTCCATCTAAAGCTAAATATTGGCGAGAGACCGATAGCGAACAAGTACAGTGATGGAAAGATGAAAAGAACTTTGAAAAGAGAGTGAAAAAGTACGTGAAATTGTTGAAAGGGAAGGGTTTGAGATCAGACTCGATATTTTGTGAGCCTTGCCTTCGTGGCGGGGTGACCCGCAGCTTATCGGGCCAGCATCGGTTTGGGCGGTAGGATAATGGCGTAGGAATGTGACTTTACTTCGGTGAAGTGTTATAGCCTGCGTTGATGCTGCCTGCCTAGACCGAGGACTGCGATTTTATCAAGGATGCTGGCATAATGATCCCAAACCGCCCGTCTTGACCACCGGACCA

>*Pichia guilliermondii* strain EXOC57B (EF 063135)

GCATATCAATAAGCGGAGGAAAAGAAACCAACAGGGATTGCCTTAGTAGCGGCGAGTGAAGCGGCAAAAGCTCAAATTTGAAATCTGGCGCCTTCGGTGTCCGAGTTGTAATTTGAAGATTGTAACCTTGGGGTTGGCTCTTGTCTATGTTTCTTGGAACAGGACGTCACAGAGGGTGAGAATCCCGTGCGATGAGATGCCCAATTCTATGTAAGGTGCTTTCGAAGAGTCGAGTTGTTTGGGAATGCAGCTCTAAGTGGGTGGTAAATTCCATCTAAAGCTAAATATTGGCGAGAGACCGATAGCGAACAAGTACAGTGATGGAAAGATGAAAAGAACTTTGAAAAGAGAGTGAAAAAGTACGTGAAATTGTTGAAAGGGAAGGGTTTGAGATCAGACTCGATATTTTGTGAGCCTTGCCTTCGTGGCGGGGTGACCCGCAGCTTATCGGGCCAGCATCGGTTTGGGCGGTAGGATAATGGCGTAGGAATGTGACTTTACTTCGGTGAAGTGTTATAGCCTGCGTTGATGCTGCCTGCCTAGACCGAGGACTGCGATTTTATCAAGGATGCTGGCATAATGATCCCAAACCGCCCGTCTTGAACCACCGGACCA

>*Pichia guilliermondii* strain EXOC14B (EF 063134)

GCATATCAATAAGCGGAGGAAAAGAAACCAACAGGGATTGCCTTAGTAGCGGCGAGTGAAGCGGCAAAAGCTCAAATTTGAAATCTGGCGCCTTCGGTGTCCGAGTTGTAATTTGAAGATTGTAACCTTGGGGTTGGCTCTTGTCTATGTTTCTTGGAACAGGACGTCACAGAGGGTGAGAATCCCGTGCGATGAGATGCCCAATTCTATGTAAGGTGCTTTCGAAGAGTCGAGTTGTTTGGGAATGCAGCTCTAAGTGGGTGGTAAATTCCATCTAAAGCTAAATATTGGCGAGAGACCGATAGCGAACAAGTACAGTGATGGAAAGATGAAAAGAACTTTGAAAAGAGAGTGAAAAAGTACGTGAAATTGTTGAAAGGGAAGGGTTTGAGATCAGACTCGATATTTTGTGAGCCTTGCCTTCGTGGCGGGGTGACCCGCAGCTTATCGGGCCAGCATCGGTTTGGGCGGTAGGATAATGGCGTAGGAATGTGACTTTACTTCGGTGAAGTGTTATAGCCTGCGTTGATGCTGCCTGCCTAGACCGAGGACTGCGATTTTATCAAGGATGCTGGCATAATGATCCCAAACCGCCCGTCTTGAACCACCGGACCAA

>*Pichia guilliermondii* strain F1004 (EU 807915)

TTCATATCCAAATAAGCGGAGGAAAAGAAACCAACAGGGATTGCCTTAGTAGCGGCGAGTGAAGCGGCAAAAGCTCAAATTTGAAATCTGGCGCCTTCGGTGTCCGAGTTGTAATTTGAAGATTGTAACCTTGGGGTTGGCTCTTGTCTATGTTTCTTGGAACAGGACGTCACAGAGGGTGAGAATCCCGTGCGATGAGATGCCCAATTCTATGTAAGGTGCTTTCGAAGAGTCGAGTTGTTTGGGAATGCAGCTCTAAGTGGGTGGTAAATTCCATCTAAAGCTAAATATTGGCGAGAGACCGATAGCGAACAAGTACAGTGATGGAAAGATGAAAAGAACTTTGAAAAGAGAGTGAAAAAGTACGTGAAATTGTTGAAAGGGAAGGGTTTGAGATCAGACTCGATATTTTGTGAGCCTTGCCTTCGTGGCGGGGTGACCCGCAGCTTATCGGGCCAGCATCGGTTTGGGCGGTAGGATAATGGCGTAGGAATGTGACTTTACTTCGGTGAAGTGTTATAGCCTGCGTTGATGCTGCCTGCCTAGACCGAGGACTGCGATTTTATCAAGGATGCTGGCATAATGATCCCAAACCGCCCGTCTTGAACCAACGG

>16

GGCGAAGTGAAGCGGCAACAGCTTCCAAATTTTGAAAGCTAGCCTTTCGGGTTCCGCATTTGTAATTTGTAGAGGATGATTTGGGGAAGCCGCCTGTCTAAGTTCCTTGGAACAGGACGTCATAGAGGGTGAGAATCCCGTATGTGACAGGAAATGGCACCCTATGTAAATCTCCTTCGACGAGTCGAGTTGTTTGGGAATGCAGCTCTAAATGGGAGGTAAATTTCTTCTAAAGCTAAATATTGGCGAGAGACCGATAGCGCACAAGTAGAGTGATCGAAAGATGAAAAGCACTTTGGAAAGAGAGTTAAAAAGCACGTGAAATTGTTGAAAGGGAAGCGCTTGCAATCAGACTTGTTTAAACTGTTCGGCCGGTCTTCTGACCGGTTTACTCAGTTTGGACAGGCCAGCATCAGTTTCGGCGGCCGGATAAAGGCTCTGGGAATGTGGCCTCCACTTCGGTGGAGGTGTTATAGCCCAGGGTGTAATACGGCCAGCCGGGACTGAGGTCCGCGCTTCGGCTAGGATGCTGGCGTAATGGTT

>*Aureobasidium pullulans* strain NS-PDC-125 (KT 922591)

ATTCGAGGAAAAGAAACCAACAGGGTTTAACCCTTAGTTCCGGCGAGTGAAGCGGCAACAGCTTCCAAATTTTGAAAGCTAGCCTTTCGGGTTCGCATTTGTAATTCTGTAGAGGATGATTTCGGGGAAGCCGCCTGTCTAAGTTCCTTCGGAACAGGACGTCATAGAGGGTGAGAATCCCGTATGTGACAGGAAATGGCACCCTATGTAAATCTCCTTCGACGAGTCGAGTTGTTTGGGAATGCAGCTCTAAATGGGAGGTAAATTTCTTCTAAAGCTAAATATTGGCGAGAGACCGATAGCGCACAAGTAGAGTGATCGAAAGATGAAAAGCACTTTGGAAAGAGAGTTAAAAAGCACGTGAAATTGTTGAAAGGGAAGCGCTTGCAATCAGACTTGTTTAAACTGTTCGGCCGGTCTTCTGACCGGTTTACTCAGTTTGGACAGGCCAGCATCAGTTTCGGCGGCCGGATAAAGGCTCTGGGAATGTGGCCTCCACTTCGGTGGAGGTGTTATAGCCCAGGGTGTAATACGGCCAAGCCGGGACTGAGGTCCGCGCTTCGGCTAGGATGCTGGCGTAATGGTTGTAAGCGACCCGTCTA

>*Aureobasidium pullulans* strain NS-PDC-139 (KT 922604)

TGAGGAAAAGAAACCAACAGGGTATGCCCTAGTAACGGCGAAGTGAAGCGGCAACAGCTTCAAATTTTGAAAGCTAGCCTTCGGGTTTCGCATTTGTAATTCTGTAGAGGATGATTTGGGGAAGCCGCCTGTCTAAGTTCCTTCGGAACAGGACGTCATAGAGGGTGAGAATCCCGTATGTGACAGGAAATGGCACCCTATGTAAATCTCCTTCGACGAGTCGAGTTGTTTGGGAATGCAGCTCTAAATGGGAGGTAAATTTCTTCTAAAGCTAAATATTGGCGAGAGACCGATAGCGCACAAGTAGAGTGATCGAAAGATGAAAAGCACTTTGGAAAGAGAGTTAAAAAGCACGTGAAATTGTTGAAAGGGAAGCGCTTGCAATCAGACTTGTTTAAACTGTTCGGCCGGTCTTCTGACCGGTTTACTCAGTTTGGACAGGCCAGCATCAGTTTCGGCGGCCGGATAAAGGCTCTGGGAATGTGGCCTCCACTTCGGTGGAGGTGTTATAGCCCAGGGTGTAATACGGCCAGCCGGGACTGAGGTCCGCGCTTCGGCTAGGATGCTGGCGTAATGGTTGTAAGCGACCCGTCTGA

>*Aureobasidium pullulans* strain NS-PDC-138 (KT 922603)

TAGGAGGAAAAGAAACCAACAGGGATTGCCCTAGTAACGGCGAGTGAAGCGGCAACAGCTCAAATTTGAAAGCTTAGCCTTCGGGTTCGCATTTGTAATTTGTAGAGGATGATTTGGGGAAGCCGCCTGTCTAAGTTCCTTGGAACAGGACGTCATAGAGGGTGAGAATCCCGTATGTGACAGGAAATGGCACCCTATGTAAATCTCCTTCGACGAGTCGAGTTGTTTGGGAATGCAGCTCTAAATGGGAGGTAAATTTCTTCTAAAGCTAAATATTGGCGAGAGACCGATAGCGCACAAGTAGAGTGATCGAAAGATGAAAAGCACTTTGGAAAGAGAGTTAAAAAGCACGTGAAATTGTTGAAAGGGAAGCGCTTGCAATCAGACTTGTTTAAACTGTTCGGCCGGTCTTCTGACCGGTTTACTCAGTTTGGACAGGCCAGCATCAGTTTCGGCGGCCGGATAAAGGCTCTGGGAATGTGGCCTCCACTTCGGTGGAGGTGTTATAGCCCAGGGTGTAATACGGCCAGCCGGGACTGAGGTCCGCGCTTCGGCTAGGATGCTGGCGTAATGGTTGTAAGCGACCCGTCTGA

>*Aureobasidium pullulans* strain NS-O-109 (KT 922828)

TAAAAGCGGAGGAAAAGAAACCAACAGGGATTGCCCTAGTAACGGCGAGTGAAGCGGCAACAGCTCAAATTTGAAAGCTAGCCTTCGGGTTCGCATTGTAATTTGTAGAGGATGATTTGGGGAAGCCGCCTGTCTAAGTTCCTTGGAACAGGACGTCATAGAGGGTGAGAATCCCGTATGTGACAGGAAATGGCACCCTATGTAAATCTCCTTCGACGAGTCGAGTTGTTTGGGAATGCAGCTCTAAATGGGAGGTAAATTTCTTCTAAAGCTAAATATTGGCGAGAGACCGATAGCGCACAAGTAGAGTGATCGAAAGATGAAAAGCACTTTGGAAAGAGAGTTAAAAAGCACGTGAAATTGTTGAAAGGGAAGCGCTTGCAATCAGACTTGTTTAAACTGTTCGGCCGGTCTTCTGACCGGTTTACTCAGTTTGGACAGGCCAGCATCAGTTTCGGCGGCCGGATAAAGGCTCTGGGAATGTGGCCTCCACTTCGGTGGAGGTGTTATAGCCCAGGGTGTAATACGGCCAGCCGGGACTGAGGTCCGCGCTTCGGCTAGGATGCTGGCGTAATGGTTGTAAGCGACCCGTCTTG

>19

GAGGAAAAGAAACCAACAGGGATTGCTCTAGTAACGGCGAGTGAAGCAGCAATAGCTCAAATTTGAAATCTGGCGTCTTCGACGTCCGAGTTGTAATTTGTAGAGGATGCTTCTGAGTGGCCACCGACCTAAGTTCCTTGGAACAGGACGTCATAGAGGGTGAGAATCCCGTATGCGGTCGGAAAGGCGCTCTATACGTAGCTCCTTCGACGAGTCGAGTTGTTTGGGAATGCAGCTCTAAATGGGAGGTAAATTTCTTCTAAAGCTAAATATTGGCCAGAGACCGATAGCGCACAAGTAGAGTGATCGAAAGATGAAAAGCACTTTGGAAAGAGAGTTAAAAAGCACGTGAAATTGTTAAAAGGGAAGGGATTGCAACCAGACTTGCTCGCGGTGTTCCGCCGGTCTTCTGACCGGTCTACTCGCCGCGTTGCAGGCCAGCATCGTCTGGTGCCGCTGGATAAGACTTGAGGAATGTAGCTCCCTCGGGAGTGTTATAGCCTCTTGTGATGCAGCGAGCGCCGGGCGAGGTCCGCGCTTCGGCTAGGATGCTGGCGTAATGGTCGTAATCCGCCCGTCTT

>*Cladosporium ramotenellum* strain H40 (KM 521834)

AAAAAGCGGAGGAAAAGAAACCAACAGGGATTGCTCTAGTAACGGCGAGTGAAGCAGCAATAGCTCAAATTTGAAATCTGGCGTCTTCGACGTCCGAGTTGTAATTTGTAGAGGATGCTTCTGAGTGGCCACCGACCTAAGTTCCTTGGAACAGGACGTCATAGAGGGTGAGAATCCCGTATGCGGTCGGAAAGGCGCTCTATACGTAGCTCCTTCGACGAGTCGAGTTGTTTGGGAATGCAGCTCTAAATGGGAGGTAAATTTCTTCTAAAGCTAAATATTGGCCAGAGACCGATAGCGCACAAGTAGAGTGATCGAAAGATGAAAAGCACTTTGGAAAGAGAGTTAAAAAGCACGTGAAATTGTTAAAAGGGAAGGGATTGCAACCAGACTTGCTCGCGGTGTTCCGCCGGTCTTCTGACCGGTCTACTCGCCGCGTTGCAGGCCAGCATCGTCTGGTGCCGCTGGATAAGACTTGAGGAATGTAGCTCCCTCGGGAGTGTTATAGCCTCTTGTGATGCAGCGAGCGCCGGGCGAGGTCCGCGCTTCGGCTAGGATGCTGGCGTAATGGTCGTAATCCGCCCGTCTTGAAACCACGGACCA

>*Cladosporium ramotenellum* strain A64 (KM 589488)

AAAAAGCGGAGGAAAAGAAACCAACAGGGATTGCTCTAGTAACGGCGAGTGAAGCAGCAATAGCTCAAATTTGAAATCTGGCGTCTTCGACGTCCGAGTTGTAATTTGTAGAGGATGCTTCTGAGTGGCCACCGACCTAAGTTCCTTGGAACAGGACGTCATAGAGGGTGAGAATCCCGTATGCGGTCGGAAAGGCGCTCTATACGTAGCTCCTTCGACGAGTCGAGTTGTTTGGGAATGCAGCTCTAAATGGGAGGTAAATTTCTTCTAAAGCTAAATATTGGCCAGAGACCGATAGCGCACAAGTAGAGTGATCGAAAGATGAAAAGCACTTTGGAAAGAGAGTTAAAAAGCACGTGAAATTGTTAAAAGGGAAGGGATTGCAACCAGACTTGCTCGCGGTGTTCCGCCGGTCTTCTGACCGGTCTACTCGCCGCGTTGCAGGCCAGCATCGTCTGGTGCCGCTGGATAAGACTTGAGGAATGTAGCTCCCTCGGGAGTGTTATAGCCTCTTGTGATGCAGCGAGCGCCGGGCGAGGTCCGCGCTTCGGCTAGGATGCTGGCGTAATGGTCGTAATCCGCCCGTCTTGACC

>*Cladosporium cf. subtilissimum* 6-2 (EU 583482)

ATATCAATAAGCGGAGGAAAAGAAACCAACAGGGATTGCTCTAGTAACGGCGAGTGAAGCAGCAATAGCTCAAATTTGAAATCTGGCGTCTTCGACGTCCGAGTTGTAATTTGTAGAGGATGCTTCTGAGTGGCCACCGACCTAAGTTCCTTGGAACAGGACGTCATAGAGGGTGAGAATCCCGTATGCGGTCGGAAAGGCGCTCTATACGTAGCTCCTTCGACGAGTCGAGTTGTTTGGGAATGCAGCTCTAAATGGGAGGTAAATTTCTTCTAAAGCTAAATATTGGCCAGAGACCGATAGCGCACAAGTAGAGTGATCGAAAGATGAAAAGCACTTTGGAAAGAGAGTTAAAAAGCACGTGAAATTGTTAAAAGGGAAGGGATTGCAACCAGACTTGCTCGCGGTGTTCCGCCGGTCTTCTGACCGGTCTACTCGCCGCGTTGCAGGCCAGCATCGTCTGGTGCCGCTGGATAAGACTTGAGGAATGTAGCTCCCTCGGGAGTGTTATAGCCTCTTGTGATGCAGCGAGCGCCGGGCGAGGTCCGCGCTTCGGCTAGGATGCTGGCGTAATGGTCGTAATCCGCCCGTCTTGAAACACG

Data for Phylogenetic tree of ITS sequences

>12

CTTCCGTAGGTGAACCTGCGGAAGGATCATTACAGTATTCTTTTGCCAGCGCTTAACTGCGCGGCGaAAAACCTTACACACAGTGTCTTTTTGATACAGAACTCTTGCTTTGGTTTGGCCTAGAGATAGGTTGGGCCAGAGGTTTAACAAAACACAATTTAATTATTTTTATTGATAGTCAAATTTTGAATTAATCTTCAAAACTTTCAACAACGGATCTCTTGGTTCTCGCATCGATGAAGAACGCAGCGAAATGCGATAAGTAATATGAATTGCAGATTTTCGTGAATCATCGAATCTTTGAACGCACATTGCGCCCTCTGGTATTCCAGAGGGCATGCCTGTTTGAGCGTCATTTCTCTCTCAAACCCCCGGGTTTGGTATTGAGTGATACTCTTAGTCGAACTAGGCGTTTGCTTGAAAAGTATTGGCATGGGTAGTACTGGATAGTGCTGTCGACCTCTCAATGTATTAGGTTTATCCAACTCGTTGAATGGTGTGGCGGGATATTTCTGGTATTGTTGGCCCGGCCTTACAACAACCAAACAAGTTTGACCTCAAATCAGGTAGGAATACCCGCTGAACTTAAGCATATCAATAAGCGGAGGAA

>*Meyerozyma caribbica* strain CBS2022 (AB 032175)

CAGTATTCTTTTGCCAGCGCTTAACTGCGCGGCGAAAAACCTTACACACAGTGTCTTTTTGATACAGAACTCTTGCTTTGGTTTGGCCTAGAGATAGGTTGGGCCAGAGGTTTAACAAAACACAATTTAATTATTTTTATTGATAGTCAAATTTTGAATTAATCTTCAAAACTTTCAACAACGGATCTCTTGGTTCTCGCATCGATGAAGAACGCAGCGAAATGCGATAAGTAATATGAATTGCAGATTTTCGTGAATCATCGAATCTTTGAACGCACATTGCGCCCTCTGGTATTCCAGAGGGCATGCCTGTTTGAGCGTCATTTCTCTCTCAAACCCCCGGGTTTGGTATTGAGTGATACTCTTAGTCGAACTAGGCGTTTGCTTGAAAAGTATTGGCATGGGTAGTACTGGATAGTGCTGTCGACCTCTCAATGTATTAGGTTTATCCAACTCGTTGAATGGTGTGGCGGGATATTTCTGGTATTGTTGGCCCGGCCTTACAACAACCAAACAAG

>*Meyerozyma caribbica* strain B-2 (AB 260137)

CAGTATTCTTTTGCCAGCGCTTAACTGCGCGGCGAAAAACCTTACACACAGTGTCTTTTTGATACAGAACTCTTGCTTTGGTTTGGCCTAGAGATAGGTTGGGCCAGAGGTTTAACAAAACACAATTTAATTATTTTTATTGATAGTCAAATTTTGAATTAATCTTCAAAACTTTCAACAACGGATCTCTTGGTTCTCGCATCGATGAAGAACGCAGCGAAATGCGATAAGTAATATGAATTGCAGATTTTCGTGAATCATCGAATCTTTGAACGCACATTGCGCCCTCTGGTATTCCAGAGGGCATGCCTGTTTGAGCGTCATTTCTCTCTCAAACCCCCGGGTTTGGTATTGAGTGATACTCTTAGTCGAACTAGGCGTTTGCTTGAAAAGTATTGGCATGGGTAGTACTGGATAGTGCTGTCGACCTCTCAATGTATTAGGTTTATCCAACTCGTTGAATGGTGTGGCGGGATATTTCTGGTATTGTTGGCCCGGCCTTACAACAACCAAACAAGT

>*Meyerozyma caribbica* strain K-3 (AB 260141)

CAGTATTCTTTTGCCAGCGCTTAACTGCGCGGCGAAAAACCTTACACACAGTGTCTTTTTGATACAGAACTCTTGCTTTGGTTTGGCCTAGAGATAGGTTGGGCCAGAGGTTTAACAAAACACAATTTAATTATTTTTATTGATAGTCAAATTTTGAATTAATCTTCAAAACTTTCAACAACGGATCTCTTGGTTCTCGCATCGATGAAGAACGCAGCGAAATGCGATAAGTAATATGAATTGCAGATTTTCGTGAATCATCGAATCTTTGAACGCACATTGCGCCCTCTGGTATTCCAGAGGGCATGCCTGTTTGAGCGTCATTTCTCTCTCAAACCCCCGGGTTTGGTATTGAGTGATACTCTTAGTCGAACTAGGCGTTTGCTTGAAAAGTATTGGCATGGGTAGTACTGGATAGTGCTGTCGACCTCTCAATGTATTAGGTTTATCCAACTCGTTGAATGGTGTGGCGGGATATTTCTGGTATTGTTGGCCCGGCCTTACAACAACCAAACAAGT

>*Meyerozyma caribbica* strain CNRMA 200700035 (EU 569003)

CAGTATTCTTTTGCCAGCGCTTAACTGCGCGGCGAAAAACCTTACACACAGTGTCTTTTTGATACAGAACTCTTGCTTTGGTTTGGCCTAGAGATAGGTTGGGCCAGAGGTTTAACAAAACACAATTTAATTATTTTTATTGATAGTCAAATTTTGAATTAATCTTCAAAACTTTCAACAACGGATCTCTTGGTTCTCGCATCGATGAAGAACGCAGCGAAATGCGATAAGTAATATGAATTGCAGATTTTCGTGAATCATCGAATCTTTGAACGCACATTGCGCCCTCTGGTATTCCAGAGGGCATGCCTGTTTGAGCGTCATTTCTCTCTCAAACCCCCGGGTTTGGTATTGAGTGATACTCTTAGTCGAACTAGGCGTTTGCTTGAAAAGTATTGGCATGGGTAGTACTGGATAGTGCTGTCGACCTCTCAATGTATTAGGTTTATCCAACTCGTTGAATGGTGTGGCGGGATATTTCTGGTATTGTTGGCCCGGCCTTACAACAACCAAACAAGT

>32

CTTCCGTAGGTGAACCTGCGGAAGGATCATTACAGTATTCTTTTGCCAGCGCTTAACTGCGCGGCGaAAAACCTTACACACAGTGTCTTTTTGATACAGAACTCTTGCTTTGGTTTGGCCTAGAGATAGGTTGGGCCAGAGGTTTAACAAAACACAATTTAATTATTTTTACAGTTAGTCAAATTTTGAATTAATCTTCAAAACTTTCAACAACGGATCTCTTGGTTCTCGCATCGATGAAGAACGCAGCGAAATGCGATAAGTAATATGAATTGCAGATTTTCGTGAATCATCGAATCTTTGAACGCACATTGCGCCCTCTGGTATTCCAGAGGGCATGCCTGTTTGAGCGTCATTTCTCTCTCAAACCCCCGGGTTTGGTATTGAGTGATACTCTTAGTCGGACTAGGCGTTTGCTTGAAAAGTATTGGCATGGGTAGTACTAGATAGTGCTGTCGACCTCTCAATGTATTAGGTTTATCCAACTCGTTGAATGGTGTGGCGGGATATTTCTGGTATTGTTGGCCCGGCCTTACAACAACCAAACAAGTTTGACCTCAAATCAGGTAGGAATACCCGCTGAACTTAAGCATATCAATAAGCGGAGGAA

>*Meyerozyma guilliermondii* strain R-14 (HM 535382)

CAGTATTCTTTTGCCAGCGCTTAACTGCGCGGCGAAAAACCTTACACACAGTGTCTTTTTGATACAGAACTCTTGCTTTGGTTTGGCCTAGAGATAGGTTGGGCCAGAGGTTTAACAAAACACAATTTAATTATTTTTACAGTTAGTCAAATTTTGAATTAATCTTCAAAACTTTCAACAACGGATCTCTTGGTTCTCGCATCGATGAAGAACGCAGCGAAATGCGATAAGTAATATGAATTGCAGATTTTCGTGAATCATCGAATCTTTGAACGCACATTGCGCCCTCTGGTATTCCAGAGGGCATGCCTGTTTGAGCGTCATTTCTCTCTCAAACCCCCGGGTTTGGTATTGAGTGATACTCTTAGTCGGACTAGGCGTTTGCTTGAAAAGTATTGGCATGGGTAGTACTAGATAGTGCTGTCGACCTCTCAATGTATTAGGTTTATCCAACTCGTTGAATGGTGTGGCGGGATATTTCTGGTATTGTTGGCCCGGCCTTACAACAACCAAACAAGTTTGACCTC

>*Meyerozyma guilliermondii* strain JCM 10735 (AB 054109)

TGCGGAAGGATCATTACAGTATTCTTTTGCCAGCGCTTAACTGCGCGGCGAAAAACCTTACACACAGTGTCTTTTTGATACAGAACTCTTGCTTTGGTTTGGCCTAGAGATAGGTTGGGCCAGAGGTTTAACAAAACACAATTTAATTATTTTTACAGTTAGTCAAATTTTGAATTAATCTTCAAAACTTTCAACAACGGATCTCTTGGTTCTCGCATCGATGAAGAACGCAGCGAAATGCGATAAGTAATATGAATTGCAGATTTTCGTGAATCATCGAATCTTTGAACGCACATTGCGCCCTCTGGTATTCCAGAGGGCATGCCTGTTTGAGCGTCATTTCTCTCTCAAACCCCCGGGTTTGGTATTGAGTGATACTCTTAGTCGGACTAGGCGTTTGCTTGAAAAGTATTGGCATGGGTAGTACTGGATAGTGCTGTCGACCTCTCAATGTATTAGGTTTATCCAACTCGTTGAATGGTGTGGCGGGATATTTCTGGTATTGTTGGCCCGGCCTTACAACAACCAAACAAGTTTGACCTCAAA

>*Meyerozyma guilliermondii* strain SMB B-G-MA4 (HM 588762)

TGCGGAAGGATCATTACAGTATTCTTTTGCCAGCGCTTAACTGCGCGGCGAAAAACCTTACACACAGTGTCTTTTTGATACAGAACTCTTGCTTTGGTTTGGCCTAGAGATAGGTTGGGCCAGAGGTTTAACAAAACACAATTTAATTATTTTTACAGTTAGTCAAATTTTGAATTAATCTTCAAAACTTTCAACAACGGATCTCTTGGTTCTCGCATCGATGAAGAACGCAGCGAAATGCGATAAGTAATATGAATTGCAGATTTTCGTGAATCATCGAATCTTTGAACGCACATTGCGCCCTCTGGTATTCCAGAGGGCATGCCTGTTTGAGCGTCATTTCTCTCTCAAACCCCCGGGTTTGGTATTGAGTGATACTCTTAGTCGGACTAGGCGTTTGCTTGAAAAGTATTGGCATGGGTAGTACTAGATAGTGCTGTCGACCTCTCAATGTATTAGGTTTATCCAACTCGTTGAATGGTGTGGCGGGATATTTCTGGTATTGTTGGCCCGGCCTTACAACAACCAAACAAGT

>*Meyerozyma guilliermondii* strain LCBG-03 (HM 991450)

TTGACTCCGCTTACTGCGCGGCGAAAACCTTACACACAGTGTCTTTTTGATACAGAACTCTTGCTTTGGTTTGGCCTAGAGATAGGTTGGGCCAAAGGTTTAACAAAACACAATTTAATTATTTTTATTGATAGTCAAATTTTGAATTAATCTTCAAAACTTTCAACAACGGATCTCTTGGTTCTCGCATCGATGAAGAACGCAGCGAAATGCGATAAGTAATATGAATTGCAGATTTTCGTGAATCATCGAATCTTTGAACGCACATTGCGCCCTCTGGTATTCCAGAGGGCATGCCTGTTTGAGCGTCATTTCTCTCTCAAACCCCCGGGTTTGGTATTGAGTGATACTCTTAGTCGAACTAGGCGTTTGCTTGAAAAGTATTGGCATGGGTAGTACTGGATAGTGCTGTCGACCTCTCAATGTATTAGGTTTATCCAACTCGTTGAATGGTGTGGCGGGATATTTCTGGTATTGTTGGCCCGGCCTTACAACAACCAAACAAGT

>13

CTTCCGTAGGTGAACCTGCGGAAGGATCATTAAAGATTGACCGAAAGGTCTTATCTCTATATCCCTCACCTCTGTGAACTGTGGACCTCCGGGTCTATTTAACAAACATCAGTGTAATGAACGTATATATCATTAAACAAAACAAAACTTTCAACAACGGATCTCTTGGCTCTCGCATCGATGAAGAACGCAGCGAAATGCGATAAGTAATGTGAATTGCAGAATTCAGTGAATCATCGAATCTTTGAACGCACCTTGCGCCTTTTGGTATTCCGAAAGGCATGCCTGTTTGAGTGTCATGAAATCTCAATCCCCCTGGGTTTATGATCTGGGTCGGACTTGGATATGGGCGTCTGCCGGTCACACGGCTCGCCTCAAATGACTTAGTGGATCTCTCTGCATCCGTGACAGACGTAATAAGTTTCGTCTTGTCCCTTGCTTATGAGTCTGCTCATAACCTGCCATCGCGCACTTtAGACTCTGACCTCAAATCAGGTAGGACTACCCGCTGAACTTAAGCATATCAATAAGCGGAGGAA

>*Cryptococcus laurentii* strain CL26 (JN 627008)

AAGATTGACCGAAAGGTCTTATCTCTATATCCCTCACCTCTGTGAACTGTGGACCTCCGGGTCTATTTAACAAACATCAGTGTAATGAACGTATATATCATTAAACAAAACAAAACTTTCAACAACGGATCTCTTGGCTCTCGCATCGATGAAGAACGCAGCGAAATGCGATAAGTAATGTGAATTGCAGAATTCAGTGAATCATCGAATCTTTGAACGCACCTTGCGCCTTTTGGTATTCCGAAAGGCATGCCTGTTTGAGTGTCATGAAATCTCAATCCCCCTGGGTTTATGATCTGGGTCGGACTTGGATATGGGCGTCTGCCGGTCACACGGCTCGCCTCAAATGACTTAGTGGATCTCTCTGCATCCGTGACAGACGTAATAAGTTTCGTCTTGTCCCTTGCTTATGAGTCTGCTCATAACCTGCCATCGCGCACTTTTAGACTCT

>*Cryptococcus laurentii* strain CBS 7140 (AY 315665)

AAGATTGACCGAAAGGTCTTATCTCTATATCCCTCACCTCTGTGAACTGTGGACCTCCGGGTCTATTTAACAAACATCAGTGTAATGAACGTATATATCATTAAACAAAACAAAACTTTCAACAACGGATCTCTTGGCTCTCGCATCGATGAAGAACGCAGCGAAATGCGATAAGTAATGTGAATTGCAGAATTCAGTGAATCATCGAATCTTTGAACGCACCTTGCGCCTTTTGGTATTCCGAAAGGCATGCCTGTTTGAGTGTCATGAAATCTCAATCCCCCTGGGTTTATGATCTGGGTCGGACTTGGAAATGGGCGTCTGCCGGTCACACGGCTCGCCTCAAATGACTTAGTGGATCTCTCTGCATCCGTGACAGACGTAATAAGTTTCGTCTTGTCCCTTGCTTATGAGTCTGCTCATAACCTGCCATCGCGCACTTTTAGACT

>*Cryptococcus laurentii* strain CL02 (JN 626984)

AAGATTGACCGAAAGGTCTTATCTCTATATCCCTCACCTCTGTGAACTGTGGACCTCCGGGTCTATTTAACAAACATCAGTGTAATGAACGTATATATCATTAAACAAAACAAAACTTTCAACAACGGATCTCTTGGCTCTCGCATCGATGAAGAACGCAGCGAAATGCGATAAGTAATGTGAATTGCAGAATTCAGTGAATCATCGAATCTTTGAACGCACCTTGCGCCTTTTGGTATTCCGAAAGGCATGCCTGTTTGAGTGTCATGAAATCTCAATCCCCCTGGGTTTATGATCTGGGTCGGACTTGGATATGGGCGTCTGCCGGTCACACGGCTCGCCTCAAATGACTTAGTGGATCTCTCTGCATCCGTGACAGACGTAATAAGTTTCGTCTTGTCCCTTGCTTATGAGTCTGCTCATAACCTGCCATCGCGCACTTTTAGACT

>*Cryptococcus laurentii* strain CL16 (JN 626998)

AAGATTGACCGAAAGGTCTTATCTCTATATCCCTCACCTCTGTGAACTGTGGACCTCCGGGTCTATTTAACAAACATCAGTGTAATGAACGTATATATCATTAAACAAAACAAAACTTTCAACAACGGATCTCTTGGCTCTCGCATCGATGAAGAACGCAGCGAAATGCGATAAGTAATGTGAATTGCAGAATTCAGTGAATCATCGAATCTTTGAACGCACCTTGCGCCTTTTGGTATTCCGAAAGGCATGCCTGTTTGAGTGTCATGAAATCTCAATCCCCCTGGGTTTATGATCTGGGTCGGACTTGGATATGGGCGTCTGCCGGTCACACGGCTCGCCTCAAATGACTTAGTGGATCTCTCTGCATCCGTGACAGACGTAATAAGTTTCGTCTTGTCCCTTGCTTATGAGTCTGCTCATAACCTGCCATCGCGCACTTTTAGACT
